# Targeting Mitochondrial PD-L1 O-GlcNAcylation to Sensitize HBV-Related HCC to Immunotherapy: Modulating Golgi-mitochondrial Crosstalk and mTOR/PGC-1α-Driven Mitochondrial Biogenesis to Overcome Resistance

**DOI:** 10.7150/ijbs.126948

**Published:** 2026-07-22

**Authors:** Ze-Bang Du, Xin-Yu Ma, Han-Yu Zhang, Jia-Ming Lei, Xin-Xin He, Wen-Qi Xu, Wen-Dan Zhou, Xiao-Gang Xia, Ao-Bo Zhuang, Xiao-Xuan Chen, You-Liang Yao, Wen-Gang Li, Yu-Chun Lin, Zhong-Ning Lin

**Affiliations:** 1 State Key Laboratory of Vaccines for Infectious Diseases, Xiang An Biomedicine Laboratory, Xiang'an Hospital of Xiamen University, National Innovation Platform for Industry-Education Integration in Vaccine Research, School of Public Health, Xiamen University, Xiamen 361102, China; 2 Department of Hepatobiliary Surgery, Cancer Research Center, Xiang'an Hospital of Xiamen University, School of Medicine, Xiamen University, Xiamen, Fujian 361102, China.

**Keywords:** HBV-related HCC, O-GlcNAcylation of mitochondrial PD-L1, mTOR/PGC-1α signaling axis, mitochondrial biogenesis, immunotherapy resistance, αPD-L1 combination therapy.

## Abstract

Hepatocellular carcinoma (HCC) remains a leading cause of cancer-related mortality worldwide, with chronic hepatitis B virus (HBV) infection representing its foremost risk factor. Although programmed death-ligand 1 (PD-L1) immune checkpoint inhibitors (ICIs) have entered clinical practice, response rates in HBV-related HCC remain limited, underscoring the urgent need for mechanism-based strategies to overcome intrinsic resistance and improve immune checkpoint blockade (ICB) efficacy by exploiting noncanonical PD-L1 functions. Here, we identify a non-canonical pathway in which the HBV-encoded X protein (HBx) drives O-GlcNAcylation (O-GlcNAc) of PD-L1 at S283 and T285, thereby promoting PD-L1 mitochondrial translocation via the GOLPH3/Drp1 axis. Mitochondrial PD-L1 (mtPD-L1) hijacks Golgi-mitochondria communication to activate the mTOR/PGC-1α axis, enhance mitochondrial biogenesis and translation, and reprogram cellular energy metabolism, ultimately conferring resistance to anti-PD-L1 antibody (αPD-L1) therapy in HBV-related HCC. Pharmacological inhibition of O-GlcNAcylation with OSMI-1 disrupts this mtPD-L1 regulatory axis, restores mitochondrial homeostasis, and sensitizes HBV-related HCC to αPD-L1 therapy. Collectively, these findings identify O-GlcNAcylated mtPD-L1 as a previously unrecognized immunometabolic checkpoint and establish the mtPD-L1-mTOR/PGC-1α axis as a key mechanism linking mitochondrial biogenesis to immunotherapy resistance. This study provides a rationale for combining αPD-L1 with OSMI-1-mediated O-GlcNAcylation inhibition as a therapeutic strategy to improve immunotherapy sensitivity in HBV-related HCC.

## Introduction

Hepatocellular carcinoma (HCC) is the sixth most frequently diagnosed cancer and the third leading cause of cancer-related death worldwide, with approximately 905,677 new cases and 830,180 deaths reported annually 1. Chronic hepatitis B virus (HBV) infection accounts for approximately 50% of HCC cases, and HBV-related HCC therefore imposes a disproportionate clinical and socioeconomic burden 2. The HBV-encoded X protein (HBx) accelerates hepatocarcinogenesis by promoting sustained proliferative signaling, immune evasion, pro-tumorigenic inflammation, and metabolic reprogramming 3, 4. Cytotoxic chemotherapy offers limited efficacy and is often associated with severe adverse effects, underscoring the urgent need for mechanism-based precision therapeutics. Immune checkpoint blockade (ICB) targeting the programmed cell death 1 (PD-1)/programmed cell death ligand 1 (PD-L1) axis has emerged as a promising therapeutic strategy 5; however, objective response rates to anti-PD-1/PD-L1 monotherapy in HBV-related HCC rarely exceed 15-30% 6. “Immune-cold” tumors, characterized by low or heterogeneous membrane PD-L1 (mPD-L1) expression together with additional immunosuppressive cues, limit T-cell recognition and effector activation 7, 8. Intracellular spatial trafficking of PD-L1 is now recognized as a key determinant of ICB efficacy 9. PD-L1 localizes not only to the plasma membrane but also to the cytoplasm, exosomes, and nucleus, with these distinct subcellular pools contributing to tumor progression and drug resistance 10. Mitochondria, as hubs of cellular metabolism and redox homeostasis, have recently been implicated in the regulation of PD-L1 trafficking and function. In triple-negative breast cancer (TNBC), paclitaxel-induced mitophagy recruits and enriches mitochondrial PD-L1 (mtPD-L1), which subsequently augments membrane PD-L1 expression and markedly enhances immune evasion 11. However, the molecular mechanisms governing PD-L1 mitochondrial translocation and its subsequent impact on mitochondrial biology remain largely unknown.

The subcellular localization and functional output of PD-L1 are tightly regulated by diverse post-translational modifications (PTMs). For example, metformin-activated AMP-activated protein kinase (AMPK) phosphorylates PD-L1 at Ser195, inducing aberrant N-glycosylation that sequesters PD-L1 in the endoplasmic reticulum for degradation and reduces its membrane abundance 12. Similarly, SIRT1-mediated deacetylation of PD-L1 promotes its interaction with HIP1R and AP2B1, driving its nuclear translocation and enhancing immune regulatory signaling in HCC cells 13. As increasing attention is directed toward non-canonical subcellular compartments, mtPD-L1 has emerged as a critical yet understudied reservoir. However, the PTM codes that govern mtPD-L1 trafficking remain largely enigmatic. O-GlcNAcylation, a reversible PTM catalyzed by O-GlcNAc transferase (OGT) and removed by O-GlcNAcase (OGA), is involved in the regulation of protein stability, folding, and subcellular inter-organelle trafficking 14, 15. Recent evidence shows that O-GlcNAcylation of phosphoglycerate kinase 1 (PGK1) at Thr255 enhances its enzymatic activity and promotes its translocation to mitochondria 16. The Golgi apparatus acts as a central hub for O-GlcNAcylation 17 and orchestrates Golgi-mitochondria communication through protein and lipid transport, mitochondrial fission, and metabolic coordination 18, 19. We therefore hypothesize that HBx-induced O-GlcNAcylation promotes PD-L1 trafficking to mitochondria via Golgi-mitochondria crosstalk, thereby reprogramming mitochondrial translation and contributing to resistance to anti-PD-L1 (αPD-L1) immunotherapy in HBV-related HCC.

Mitochondria, as the cellular energy-producing organelles, depend on both mitochondrial quality control and continuous mitochondrial biogenesis to meet the dynamic metabolic demands of the cell 20. This process is coordinated by nuclear and mitochondrial genomes, encompassing mitochondrial DNA (mtDNA) replication, protein synthesis, membrane assembly, and the formation of functional respiratory complexes 21. Emerging evidence highlights the pathophysiological relevance of mitochondrial biogenesis. HBx enhances mitochondrial biogenesis, resulting in elevated cytochrome c oxidase activity, enhanced mitochondrial membrane potential, increased mitochondrial reactive oxygen species (mtROS) generation, upregulated cyclooxygenase-2 (COX-2) expression, and accelerated HCC cell proliferation 22. Similarly, febrile conditions enhance mitochondrial mass and function in CD8^+^ T-cells via accelerated mitochondrial translation 23, underscoring the central role of mitochondrial translation in generating functional mitochondria during biogenesis 24. Given the pivotal role of mitochondrial biogenesis in cellular metabolic reprogramming and the emerging repertoire of mtPD-L1 functions, we hypothesize that mtPD-L1 controls mitochondrial biogenesis—specifically via mitochondrial translation—in HBx-expressing HCC cells. Dissecting this mechanism may offer a novel conceptual framework for understanding the non-immune functions of mtPD-L1 in HBV-related HCC.

In this study, we demonstrate that HBV-related HCC is characterized by mtPD-L1-driven enhancement of mitochondrial biogenesis, a process dependent on HBx-induced O-GlcNAcylation. Mechanistically, O-GlcNAcylated PD-L1 accumulates in mitochondria via Golgi**-**mitochondria crosstalk, where it activates the mTOR/PGC-1α axis to enhance mitochondrial translation and energetic output. This mtPD-L1-dependent pathway contributes to immune resistance and tumor progression, even in the presence of αPD-L1 therapy. Notably, pharmacological inhibition of O-GlcNAcylation with OSMI-1 restores immunotherapeutic sensitivity and suppresses tumor growth. Collectively, our findings uncover a previously unrecognized role of mtPD-L1 in modulating mitochondrial function and immune evasion, offering a rationale for combining αPD-L1 with OGT inhibition to improve ICB efficacy in HBV-related HCC.

## Materials and methods

Additional materials and methods are described in the Supplementary files.

### Bioinformatics analysis

Public datasets, including datasets from the Gene Expression Omnibus (GEO) and The Cancer Genome Atlas (TCGA) liver hepatocellular carcinoma (LIHC) dataset, were used to extract RNA-expression data from HCC patients. Dataset GSE279750 contains HCC samples from 10 patients (4 non-responders and 6 responders) treated with αPD-L1 therapy. Dataset GSE222281 comprises MHCC-97H and MHCC-97L cells stably expressing Flag-OGT. Details of additional GEO datasets are listed in Supplementary Table S1. Correlation analysis between *CD274* expression and mitochondrial biogenesis-related genes was performed using Pearson correlation analysis.

### HCC tissue specimens

Six HCC patients who underwent surgical resection at Xiang'an Hospital of Xiamen University (Xiamen, China) were enrolled, including three HBV-positive and three HBV-negative cases. Tumor tissues and matched adjacent non-tumorous tissues were collected from each patient. Fresh specimens were snap-frozen in liquid nitrogen immediately after resection and stored at -80 °C for subsequent molecular analyses. Parallel tissue samples were fixed, paraffin-embedded, and subjected to histopathological evaluation. The expression of PD-L1, PGC-1α, TFAM, and NRF1 was assessed by immunohistochemistry (IHC). In addition, paraffin-embedded tumor and matched adjacent non-tumorous tissue sections were collected from 10 HBV-positive HCC patients who had previously received ICB therapy at the same institution. IHC was performed to assess PD-L1, global O-GlcNAc, PGC-1α, and mTOR expression.

Antibody details are listed in Supplementary Table S2. All patients provided written informed consent. The study protocol was approved by the Ethics Committee of Xiamen University and conducted in accordance with the Declaration of Helsinki.

### HBx transgenic (HBx-Tg) mouse study

HBx-Tg mice were generated as previously described 25. HBx-Tg and wild-type (WT) mice were maintained for up to 18 months. Cohorts (n = 5 per group) were euthanized at 6-, 12-, and 18-month intervals. Harvested liver tissues were processed for lysate preparation, serial cryosectioning, and multiparametric analyses, including immunofluorescence (IF) for PD-L1 and TOM20. Transcriptomic analyses were performed on HBx-Tg (n = 4) and WT (n = 4) livers 26. DEGs were identified (|log_2_FC| ≥ 1, *P* < 0.05) and subjected to profiling focused on Golgi and mitochondrial translation, and to GSEA.

All animal procedures were approved by the Experimental Animal Ethics Committee of Xiamen University (approval No. XMULAC20220282, dated 12 March 2022).

### *In vivo* subcutaneous tumor model

HBx-Tg mice (8-12 weeks old) were maintained under specific pathogen-free conditions as previously described 26. Mice were randomly assigned to four groups (n = 5 per group) and treated for 3 weeks as follows: vehicle control (Ctrl), OSMI-1 (1 mg/kg, i.p., every 3 days), αPD-L1 (5 mg/kg, i.p., every 3 days), or OSMI-1 plus αPD-L1.

CD8^+^ T-cell depletion and xenograft model: CD8^+^ T-cell depletion was achieved by an initial i.p. injection of 200 μg anti-mouse CD8α (InVivoMAb, Bio X Cell; West Lebanon, NH, USA) 2 days before tumor inoculation, followed by maintenance dosing every 4 days. Mice were then subcutaneously inoculated in the right flank with 5 × 10⁶ human tumor cells (MHCC-97H) suspended in 100 μL PBS. Tumor volume and body weight were monitored every 3 days. Mice were euthanized when tumors reached 1000 mm^3^ or developed ulceration. Excised tumors were weighed and processed for subsequent analyses.

Syngeneic subcutaneous model: Mice were inoculated subcutaneously in the right flank with 5 × 10⁶ murine tumor cells (Hepa1-6) suspended in 100 μL PBS. Tumor growth and body weight were monitored every 3 days. Mice were euthanized when tumors reached 1000 mm³ or developed ulceration. Tumors were harvested, weighed, and subjected to further analysis.

All animal procedures were approved by the Experimental Animal Ethics Committee of Xiamen University (approval No. XMULAC20220282, dated 12 March 2022).

### Mouse CD8⁺ T-cell isolation and non-contact soluble-factor response assay

OT-1 mice were purchased from GemPharmatech Co., Ltd. (Nanjing, Jiangsu, China). CD8⁺ T-cells were isolated from OT-1 splenocytes by negative magnetic selection (Biosharp, Beijing, China). Purified cells were cultured in RPMI-1640 complete medium supplemented with 10% fetal bovine serum (FBS) and 50 μM β-mercaptoethanol at 37 °C in a humidified atmosphere containing 5% CO₂, and stimulated with anti-CD3/anti-CD28-coated beads at a 1:1 ratio (Biosharp). IL-2 was added as required to support CD8⁺ T-cell expansion and maintenance.

For the non-contact Transwell soluble-factor response assay, anti-CD3/CD28-preactivated OT-1-derived CD8⁺ T-cells were placed in the upper chamber, while HBx-Hepa1-6 cells were seeded in the lower chamber. HBx-Hepa1-6 cells do not express OVA, and neither OVA protein nor SIINFEKL peptide was added. Thus, this assay was designed to assess tumor-cell responses to soluble factors released by anti-CD3/CD28-preactivated CD8⁺ T-cells, rather than OVA/ SIINFEKL-H-2Kᵇ-restricted OT-1 TCR-mediated recognition or tumor-cell killing. Where indicated, co-cultures were treated with αPD-L1 and/or OSMI-1. Tumor-cell responses were evaluated using an LDH assay kit (Beyotime, Shanghai, China) to assess cell injury, and cytokine secretion was quantified using IFN-γ and TNF-α ELISA kits. All readouts were interpreted within the context of an anti-CD3/CD28-preactivated CD8⁺ T-cell-derived soluble effector environment, without antigen-specific TCR engagement.

### Cytokine quantification

Cell culture supernatants were analyzed in triplicate using commercial ELISA kits for human/mouse IFN-γ (Yisheng Biotechnology, Hangzhou, Zhejiang, China) and TNF-α (Linko Biotechnology, Hangzhou, Zhejiang, China) according to the manufacturers' protocols. Absorbance was measured at 450 nm on a CLARIOstar microplate reader (BMG Labtech, Offenburg, Germany).

### Co-immunoprecipitation (Co-IP) and LC-MS/MS

HepG2.2.15 and MHCC-97H cells were lysed in ice-cold RIPA buffer supplemented with protease inhibitors; lysates were clarified (10,000 × g, 10 min, 4 °C) and incubated with antibodies against GOLPH3, Drp1, O-GlcNAc, OGT, or His for 2 h at 4 °C, followed by incubation with Protein A/G magnetic beads for an additional 2 h at 4 °C. Immunocomplexes were washed, eluted, and analyzed by immunoblotting and silver staining.

For interactome analysis, Co-IP eluates were separated by sodium dodecyl sulfate-polyacrylamide gel electrophoresis (SDS-PAGE), visualized by Coomassie Brilliant Blue, and subjected to in-gel tryptic digestion. Peptides were analyzed by LC-MS/MS at Applied Protein Technology (Shanghai, China).

### Molecular docking analysis

The protein structures used for molecular docking were PD-L1 (UniProt ID: Q9NZQ7) and OGT (UniProt ID: O15294). Site-directed mutations in PD-L1 were introduced using PyMOL. Protein-protein docking was performed with the HDOCK server (http://hdock.phys.hust.edu.cn/). Before docking, protein structures were preprocessed in PyMOL 2.4 by removing water molecules and redundant ligands and by adding hydrogen atoms.

Docking scores, confidence scores, and ligand RMSD values were used to evaluate docking performance. The number of docking poses was set to 10, and the model with the highest docking score was selected as the optimal complex for subsequent analysis. The final docking results were visualized in PyMOL 2.4 to characterize the interaction interface and binding mode between PD-L1 and OGT.

### Proximity ligation assay (PLA)

Proximity between GOLPH3 and Drp1, as well as between PD-L1 and OGT, was assessed using the Duolink® *In Situ* Detection Reagents (Sigma-Aldrich, St. Louis, MO, USA) according to the manufacturer's protocol 27. Briefly, cells (5 × 10⁴ per well) were seeded onto sterile coverslips, fixed with 4% paraformaldehyde, permeabilized with 0.5% Triton X-100, and blocked with Duolink II Blocking Solution for 1 h. Samples were then incubated overnight at 4 °C with primary antibodies against the indicated targets, followed by incubation with Duolink PLA anti-rabbit PLUS and anti-mouse MINUS probes. After washing, ligation and rolling-circle amplification were performed at 37 °C for 30 and 100 min, respectively. PLA fluorescent signals were imaged using a Leica SP8 confocal microscope and quantified with Image-Pro Plus v6.0.

### Metabolic flux analysis

Equal numbers of cells were incubated with [U-^13^C] glucose for the indicated times. After washing three times with PBS, metabolites were extracted in 1 mL ice-cold methanol/acetonitrile/water (2:2:1, v/v/v). Cells were scraped into the extraction solvent, vortexed for 30 s, and sonicated on ice for 10 min. The lysates were snap-frozen in liquid nitrogen, thawed, and sonicated again for 10 min; this cycle was repeated three times. Proteins were precipitated at -20 ℃ for 1 h, and supernatants were collected after centrifugation (13,000 × g, 15 min, 4 ℃). An 800-μL aliquot was dried under vacuum, reconstituted in LC-MS-grade water, and subjected to LC-MS/MS analysis.

### Mitochondrial nascent-protein labeling (AHA assay)

*De novo* mitochondrial translation was monitored using the L-azidohomoalanine (AHA) fluorescent labeling assay 28. Cells were first pre-incubated in methionine-free medium for 30 min, then labeled with 50 μM AHA working solution (Click-iT^TM^, Thermo Fisher) for 4 h. After three washes with PBS, cells were fixed with 4 % paraformaldehyde (30 min, RT), permeabilized with 0.25 % Triton X-100 for 10 min, blocked with 1 % BSA in PBS for 1 h, and subjected to the Click-iT® reaction (30 min, RT, protected from light). Cells were incubated with a primary anti-TOM20 antibody overnight at 4 ℃, followed by incubation with a fluorophore-conjugated secondary antibody (1:500, 1 h, RT, protected from light). Nuclei were counterstained with DAPI (Beyotime).

### Statistical analysis

All quantitative data are presented as mean ± standard deviation (SD). Statistical analyses were performed using GraphPad Prism 8.0 (GraphPad Software, San Diego, CA, USA). Two-group comparisons were carried out with an unpaired two-tailed Student's *t*-test. Comparisons among multiple groups were analyzed by one-way analysis of variance (ANOVA) followed by Tukey's post-hoc test. *P* values < 0.05 were considered statistically significant.

## Results

### HBx-driven mtPD-L1 orchestrates resistance to αPD-L1-associated immune pressure in HCC

**Figure 1:**
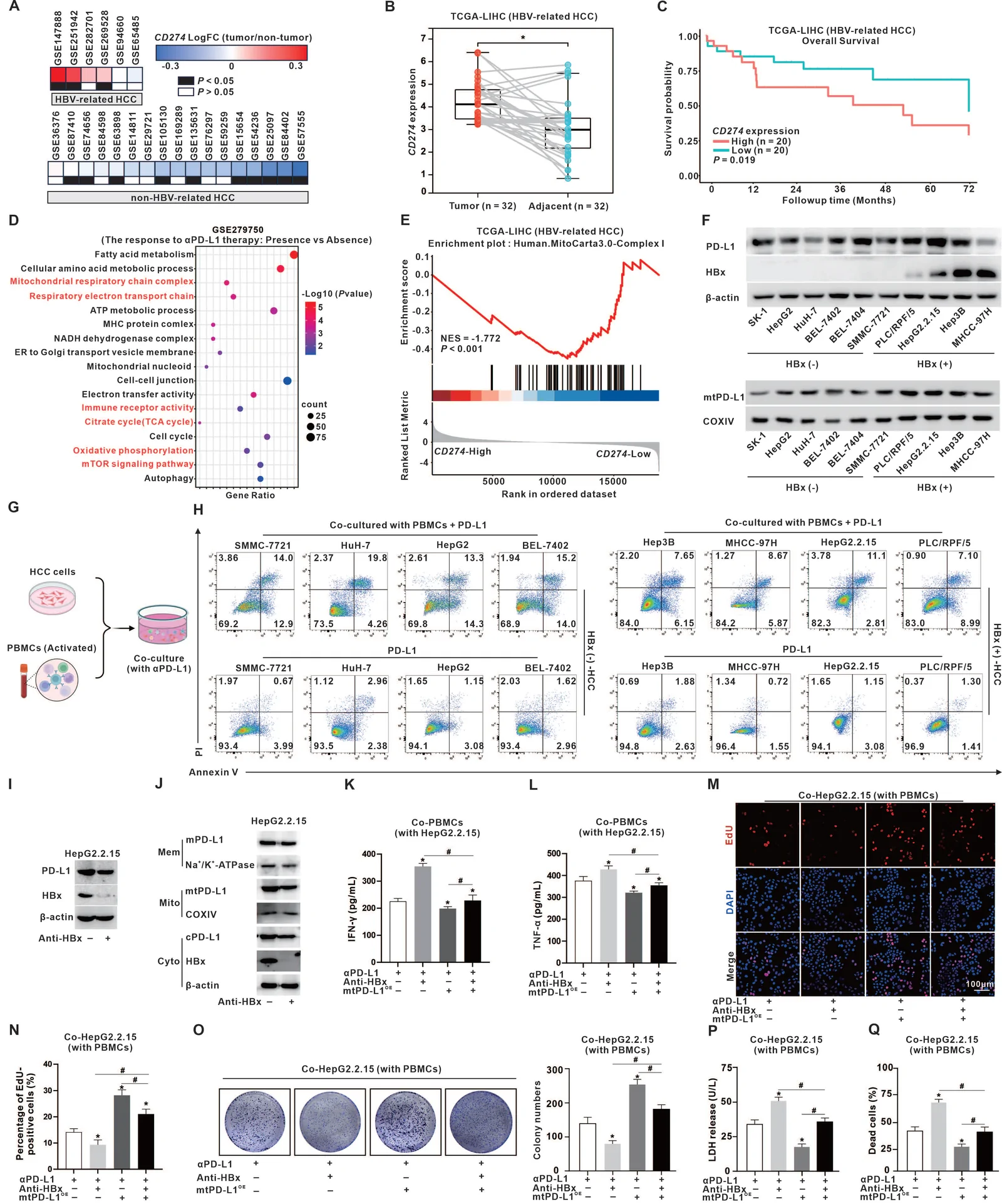
** HBx-driven mtPD-L1 orchestrates resistance to αPD-L1-associated immune pressure in HCC.** (A) Twenty-three GEO datasets from HBV-related HCC cohorts were integrated. Large-scale data mining categorized samples as HBV(+) HCC or HBV(-) HCC cases. *CD274* (encoding PD-L1) mRNA expression was compared between HCC tumors and adjacent non-tumor tissues. (B) Paired analysis of *CD274* mRNA expression in HBV-related HCC using TCGA-LIHC matched samples (tumor, n = 32; adjacent non-tumor, n = 32). Matched pairs are connected by lines to visualize within-patient variation. (C) Kaplan-Meier overall survival (OS) curves stratifying HBV-related TCGA-LIHC patients by *CD274*-high (top 50%, n = 20) versus *CD274*-low (bottom 50%, n = 20) expression. (D) GO enrichment of differentially expressed genes (DEGs) between responders (n = 6) and non-responders (n = 4) to anti-PD-L1 (αPD-L1) therapy in HCC patients from the GEO dataset GSE279750. (E) GSEA of DEGs between *CD274*-high and *CD274*-low HBV-related HCC samples from TCGA-LIHC. (F) Expression levels of total PD-L1 (upper) and mtPD-L1 (lower) were detected by Western blotting (WB) in representative HBV(-) and HBV(+) HCC cell lines. (G) Schematic of the co-culture system. (H) Representative flow-cytometric plots of apoptosis in HCC cell lines co-cultured with activated PBMCs and treated with or without αPD-L1 (10 μg/mL, 24 h). (I-J) HepG2.2.15 cells were transfected with or without the anti-HBx PB-20F3 transposon plasmid for 24 h. (I) Total PD-L1 levels were detected by WB. (J) PD-L1 levels in subcellular fractions (membrane, mitochondria, and cytosol) were detected by WB. (K-Q) HepG2.2.15 cells overexpressing mitochondria-targeted PD-L1 (mtPD-L1^OE^), generated by fusing PD-L1 to a mitochondrial targeting sequence (MTS), were co-cultured with activated PBMCs and treated with or without αPD-L1 (10 μg/mL) and the anti-HBx PB-20F3 transposon plasmid for 24 h. (K-L) PBMC-derived IFN-γ (K) and TNF-α (L) levels were measured by ELISA. (M) Representative images of EdU incorporation assays. Scale bar: 100 μm. (N) Quantification of EdU-positive cells. (O) Representative images (left) and quantification (right) of colony formation. (P-Q) LDH release (P) and dead-cell percentage (Q). Data are mean ± SD, n = 3. *, *P* < 0.05 versus control; #, *P* < 0.05 versus the indicated group.

To dissect the mechanistic role of PD-L1 in HBV-related HCC, we first performed an integrated analysis of 23 GEO-derived HBV-related HCC cohorts. Large-scale data mining revealed consistent upregulation of *CD274* (encoding PD-L1) in most HBV-positive HCC tumors, whereas non-HBV cohorts showed heterogeneous or even reduced expression patterns (Fig. 1A). To ensure a rigorous paired comparison, we further re-filtered the TCGA-LIHC cohort and analyzed 32 matched tumor-adjacent tissue pairs. This paired analysis showed that *CD274* mRNA levels were significantly higher in HBV-related HCC tumors than in the corresponding adjacent liver tissues (Fig. 1B). Kaplan-Meier analysis of the TCGA-LIHC cohort further demonstrated that low *CD274* expression was specifically associated with poorer overall survival (OS) in HBV-positive patients (Fig. 1C). Consistently, receiver operating characteristic (ROC) curve analysis supported the diagnostic value of *CD274* expression in distinguishing HBV-associated tumors from adjacent tissues in the TCGA-LIHC cohort (Fig. S1A). Collectively, these findings identify PD-L1 as a key molecular feature of HBV-related HCC with both pathogenic and prognostic relevance.

We next stratified HCC patients by response to αPD-L1 immunotherapy and performed gene-set enrichment analysis (GSEA) of differentially expressed genes (DEGs) in the GSE279750 dataset. Compared with non-responders, responders showed significant enrichment in "mitochondrial respiratory chain complexes", "tricarboxylic acid (TCA) cycle", "immune receptor activity", and "mTOR signaling pathway" (Fig. 1D and Fig. S1B). Consistently, independent GO and GSEA analyses of tumors with high versus low *CD274* expression in HBV-positive patients from the TCGA-LIHC cohort revealed enrichment of "regulation of mitochondrial organization", "protein localization to mitochondrion", and "trans-Golgi network transport" (Fig. S1C-D), as well as alterations in the "mitochondrial complex I pathway" (Fig. 1E). Together, these findings indicate that PD-L1-associated mitochondrial regulation is closely linked to the efficacy of αPD-L1 immunotherapy in HCC.

To further investigate this relationship *in vivo*, we employed the HBx-Tg mouse model. Immunoblotting showed progressive increases in both total and mitochondrial PD-L1 in HBx-Tg mice compared with wild-type (WT) controls (Fig. S1E-F), and immunofluorescence (IF) further confirmed the progressive localization of mitochondrial PD-L1 (mtPD-L1) in HBx-Tg mouse livers (Fig. S1G). Consistently, analysis of ten HCC cell lines showed that HBx-positive cells expressed higher levels of total and mtPD-L1 than HBx-negative cells; among the HBx-positive lines, HepG2.2.15 cells exhibited higher total and mtPD-L1 levels than MHCC-97H cells (Fig. 1F). In co-culture assays, all groups were processed in parallel and analyzed within a unified statistical framework. In addition to the PBMC-tumor co-culture plus αPD-L1 condition, a matched tumor-only plus αPD-L1 control was included to exclude direct antibody effects on tumor cells. Under these conditions, αPD-L1-induced tumor killing was significantly greater in the PBMC-tumor co-culture group than in the tumor-only control, indicating that tumor-cell suppression depended mainly on PBMC-associated effector activity rather than a direct effect of αPD-L1 on tumor cells. Notably, this immune killing was partially attenuated in HBx-positive HCC cells compared with HBx-negative counterparts, consistent with HBx-associated resistance to αPD-L1-mediated immune pressure (Fig. 1G-H). Collectively, these findings indicate that HBx promotes PD-L1 upregulation and mitochondrial localization, thereby reducing the susceptibility of HCC cells to αPD-L1-associated immune effector pressure.

Building on our previous finding that mtPD-L1 regulates tumor-cell behavior 29, we examined PD-L1 subcellular distribution in HBx-expressing models. HBx overexpression in HepG2 cells markedly increased total PD-L1 levels (Fig. S1H), whereas HBx silencing in HepG2.2.15 cells using an anti-HBx transposon plasmid significantly reduced PD-L1 expression (Fig. 1I). Subcellular fractionation showed that HBx preferentially increased mtPD-L1 (Fig. S1I), while anti-HBx treatment decreased mtPD-L1 in an HBx-dependent manner (Fig. 1J). By contrast, membrane-associated PD-L1 (mPD-L1) changed only modestly, and cytosolic PD-L1 (cPD-L1) remained largely unchanged (Fig. 1J and Fig. S1I). These findings indicate that HBx drives compartment-specific redistribution of PD-L1, with mitochondrial accumulation potentially contributing to resistance to PD-L1 blockade. To further define the functional role of mtPD-L1, we engineered HBx-expressing HCC cells to overexpress mitochondria-targeted PD-L1 (mtPD-L1^OE^) by fusing PD-L1 to a mitochondrial targeting sequence (MTS) (Fig. S1J). In co-culture with activated PBMCs, combined anti-HBx and αPD-L1 treatment synergistically increased PBMC-derived IFN-γ and TNF-α secretion (Fig. 1K-L), and markedly inhibited proliferation, colony formation (Fig. 1M-O), and viability of HBx-expressing HCC cells, while increasing LDH release and cell death (Fig. 1P-Q). In contrast, enforced mtPD-L1 expression largely reversed these effects, attenuating PBMC-derived cytokine release (Fig. S1K-L) and preserving tumor cell survival despite αPD-L1 treatment (Fig. S1M-Q). Together, these results identify HBx-driven mtPD-L1 as a critical mediator of resistance to αPD-L1-associated immune pressure in HBV/HBx-related HCC.

### O-GlcNAcylation of PD-L1 drives its mitochondrial translocation via remodeling Golgi-mitochondria communication in HBx-expressing HCC cells

**Figure 2:**
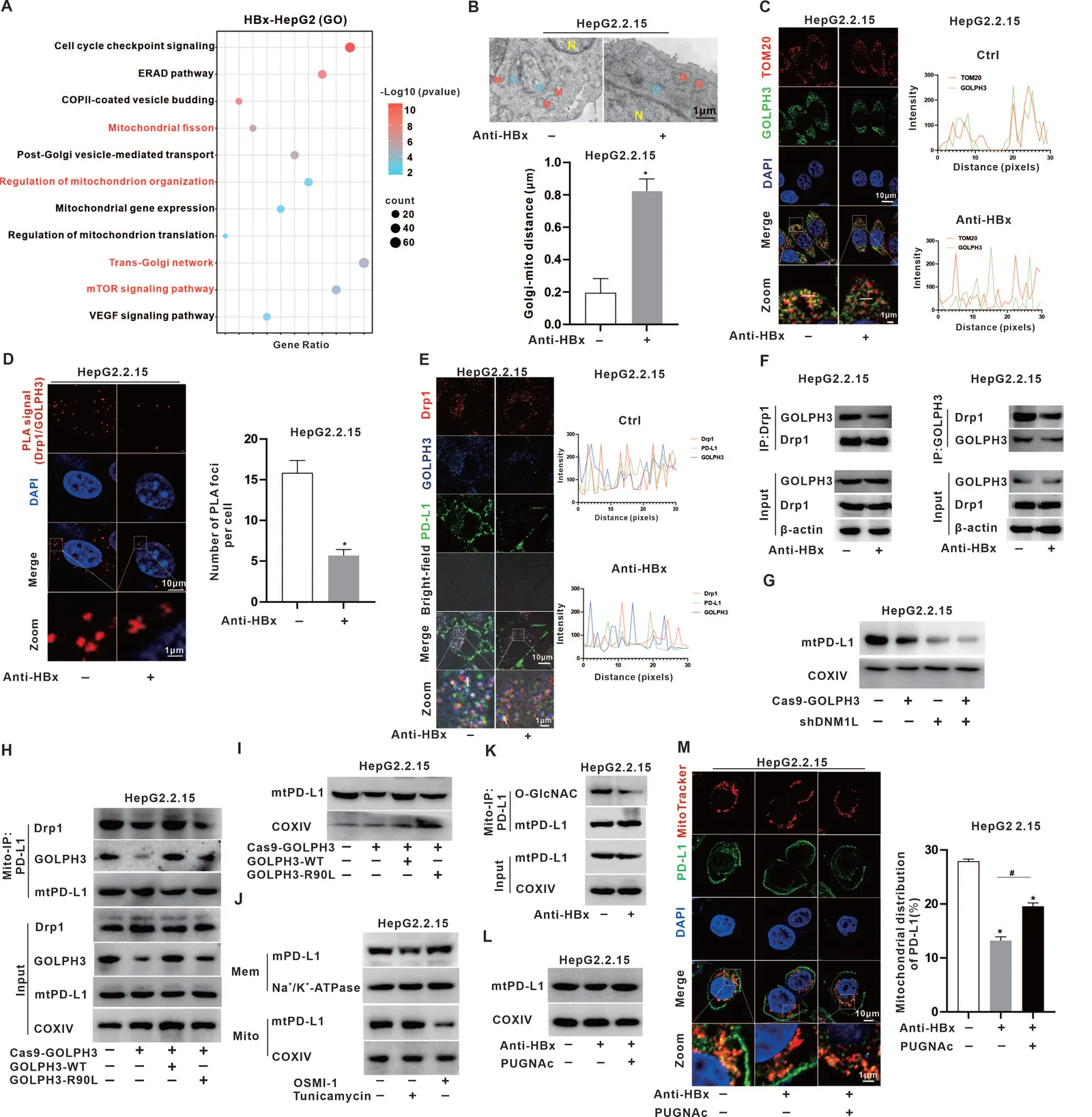
** O-GlcNAcylation of PD-L1 drives its mitochondrial translocation via remodeling Golgi-mitochondria communication in HBx-expressing HCC cells.** (A) RNA-sequencing transcriptomic analysis was performed in HBx-expressing HepG2 cells (n = 3), followed by Gene Ontology (GO) enrichment analysis of DEGs. (B) Representative TEM images showing the proximity between the Golgi apparatus and mitochondria in HepG2.2.15 cells treated with anti-HBx (upper); quantification of the shortest Golgi-mitochondria distance (μm) is shown in the bar graphs (lower). G, Golgi apparatus; M, mitochondrion; N, nucleus. Scale bar: 1 μm. (C) Representative immunofluorescence (IF) images showing TOM20 (mitochondrial marker, green) and GOLPH3 (Golgi marker, red) co-staining in anti-HBx-treated HepG2.2.15 cells. Nuclei were counterstained with DAPI (blue) (left); line-scan intensity profiles are shown to illustrate signal co-distribution (right). Scale bar: 10 μm (main) and 1 μm (Zoom). (D-F) HepG2.2.15 cells were transfected with or without the anti-HBx PB-20F3 transposon plasmid for 24 h. (D) *In situ* PLA detecting endogenous GOLPH3-Drp1 proximity. PLA puncta (red) indicate GOLPH3-Drp1 molecular proximity; nuclei were counterstained with DAPI (blue). Representative images and enlarged insets (Zoom) are shown (left); quantification of PLA foci per cell is shown (right). Scale bar: 10 μm (main) and 1 μm (Zoom). (E) Representative IF images showing Drp1 (red), GOLPH3 (blue), and PD-L1 (green), together with bright-field and merged views. Insets show higher-magnification regions (Zoom) (left); line-scan intensity profiles are shown (right). Scale bar: 10 μm (main) and 1 μm (Zoom). (F) Reciprocal Co-IP validating the GOLPH3-Drp1 association. (G) WB analysis of mtPD-L1 in mitochondrial fractions from HepG2.2.15 cells subjected to CRISPR/Cas9-mediated GOLPH3 disruption (Cas9-GOLPH3) and/or shRNA-mediated *DNM1L* knockdown (shDNM1L; Drp1-KD). (H-I) CRISPR/Cas9-mediated GOLPH3 knockdown (Cas9-GOLPH3) was established in HepG2.2.15 cells, followed by transfection with wild-type (GOLPH3-WT) or R90L mutant (GOLPH3-R90L) plasmids for 24 h. (H) Mito-IP assessing the interaction between mtPD-L1 and the GOLPH3/Drp1 complex. (I) mtPD-L1 levels in mitochondrial fractions were detected by WB. (J) HepG2.2.15 cells were treated for 24 h with the O-GlcNAcylation inhibitor OSMI-1 (10 μM) or the N-glycosylation inhibitor tunicamycin (TM, 1 μg/mL). Membrane and mitochondrial PD-L1 levels were then detected by WB. (K) Mito-IP assessing the O-GlcNAcylation status of mtPD-L1. (L-M) Anti-HBx-treated HepG2.2.15 cells were treated for 24 h with the O-GlcNAcase (OGA) inhibitor PUGNAc (10 μM). (L) mtPD-L1 levels in mitochondrial fractions. (M) Representative IF images showing mtPD-L1 (green) localization relative to mitochondria (MitoTracker, red). Nuclei were counterstained with DAPI (blue) (left); line-scan intensity profiles are shown to illustrate signal co-distribution (right). Scale bar: 10 μm (main) and 1 μm (Zoom). Data are mean ± SD, n = 3. *, *P* < 0.05 versus control; #, *P* < 0.05 versus the indicated group.

To elucidate the mechanism underlying enhanced mtPD-L1 localization in HBx-expressing HCC cells, we interrogated transcriptomic data from HBx-expressing HepG2 cells and HBx-Tg mouse livers. GO enrichment analyses of both datasets revealed significant enrichment of terms related to "trans-Golgi network (TGN)", "mTOR signaling pathway", and "mitochondrial organization/fission" (Fig. 2A and Fig. S2A-C), suggesting that HBx remodels the spatial and functional interface between the Golgi apparatus and mitochondria. Consistently, high-resolution TEM revealed perinuclear clustering of mitochondria adjacent to the Golgi in HBx-expressing HepG2 cells and HepG2.2.15 cells, and quantitative analysis further demonstrated a reduced a shorter minimum Golgi-mitochondria distance in both models (Fig. 2B and Fig. S2D). To further assess organelle proximity, we labeled the TGN and mitochondria with GOLPH3 and TOM20, respectively. IF analysis showed increased GOLPH3-TOM20 proximity in HBx-expressing HepG2 cells, characterized by partial and site-restricted apposition rather than uniform co-localization, as further supported by magnified views and line-scan profiling (Fig. S2E). Conversely, intracellular neutralization of HBx in HepG2.2.15 cells increased the Golgi-mitochondria distance and attenuated GOLPH3-TOM20 proximity (Fig. 2C). Collectively, these data indicate that HBx promotes regulated Golgi-mitochondria proximity/contact, thereby enhancing inter-organelle communication.

Previous studies have shown that PI(4)P-enriched Golgi-derived vesicles recruit GOLPH3 together with Drp1 to regulate mitochondrial dynamics 30. We therefore hypothesized that the GOLPH3/Drp1 complex mediates PD-L1 trafficking to mitochondria. *In situ* PLA and IF analyses demonstrated endogenous GOLPH3-Drp1 proximity in intact HepG2.2.15 cells, whereas intracellular HBx neutralization markedly reduced the PLA signal and attenuated their spatial apposition (Fig. 2D-E), indicating that HBx promotes assembly of the endogenous GOLPH3/Drp1 complex. This was further corroborated by reciprocal endogenous Co-IP, which showed reduced GOLPH3-Drp1 co-precipitation following anti-HBx treatment (Fig. 2F). Consistent with this, PD-L1 exhibited robust association with both GOLPH3 and Drp1, and anti-HBx treatment diminished PD-L1 proximity to these proteins, placing HBx upstream of PD-L1 engagement with the GOLPH3/Drp1 machinery. Functionally, depletion of either GOLPH3 or Drp1 reduced mtPD-L1 abundance, whereas co-silencing of both nearly abolished mtPD-L1 enrichment (Fig. 2G). Consistently, mitochondria-targeted immunoprecipitation (Mito-IP) revealed that PD-L1 association with the GOLPH3/Drp1 complex was diminished upon GOLPH3 knockdown, restored by wild-type GOLPH3, but not by the PI(4)P-binding-deficient GOLPH3-R90L mutant (Fig. 2H and Fig. S2F). Concomitantly, re-expression of wild-type, but not R90L, GOLPH3 restored mtPD-L1 levels (Fig. 2I and Fig. S2G). Collectively, these data support a model in which engagement of the GOLPH3/Drp1 complex is required for efficient mitochondrial enrichment of PD-L1, rather than representing a secondary interaction after PD-L1 has already reached mitochondria.

The Golgi apparatus is a central hub for protein glycosylation, including N-glycosylation and O-GlcNAcylation, which regulate protein folding, trafficking, and subcellular localization 31. To determine whether glycosylation directs PD-L1 mitochondrial trafficking, HBx-expressing HCC cells were treated with tunicamycin (TM), an inhibitor of N-glycosylation, or OSMI-1, an inhibitor of O-GlcNAcylation. TM markedly reduced mPD-L1, supporting a requirement for N-glycosylation in plasma-membrane localization. In contrast, OSMI-1 selectively decreased mtPD-L1 without affecting mPD-L1 (Fig. 2J and Fig. S2H), indicating that O-GlcNAcylation specifically governs PD-L1 mitochondrial translocation. Co-IP further showed that the interaction between PD-L1 and O-GlcNAc transferase (OGT) was enhanced in HBx-expressing HepG2 cells and reduced in HepG2.2.15 cells following anti-HBx treatment (Fig. S2I). Consistently, Mito-IP demonstrated increased O-GlcNAcylation of mtPD-L1 in HBx-expressing HCC cells (Fig. S2J), which was attenuated by anti-HBx treatment (Fig. 2K). Functionally, OSMI-1 significantly reduced mtPD-L1 abundance, whereas the O-GlcNAcase (OGA) inhibitor PUGNAc increased it (Fig. 2L and Fig. S2K). Mechanistically, Mito-IP showed that OSMI-1 disrupted, whereas PUGNAc enhanced, the interaction between mtPD-L1 and the GOLPH3/Drp1 complex (Fig. 2M and Fig. S2L-M). Together, these findings demonstrate that HBx-driven O-GlcNAcylation enables PD-L1 engagement with the GOLPH3/Drp1 shuttle, thereby promoting its mitochondrial translocation through remodeled Golgi-mitochondria communication in HBx-expressing HCC cells.

### OGT-mediated O-GlcNAcylation of PD-L1 facilitates its mitochondrial localization and promotes immune pressure-resistant phenotypes in HepG2.2.15 cells

**Figure 3:**
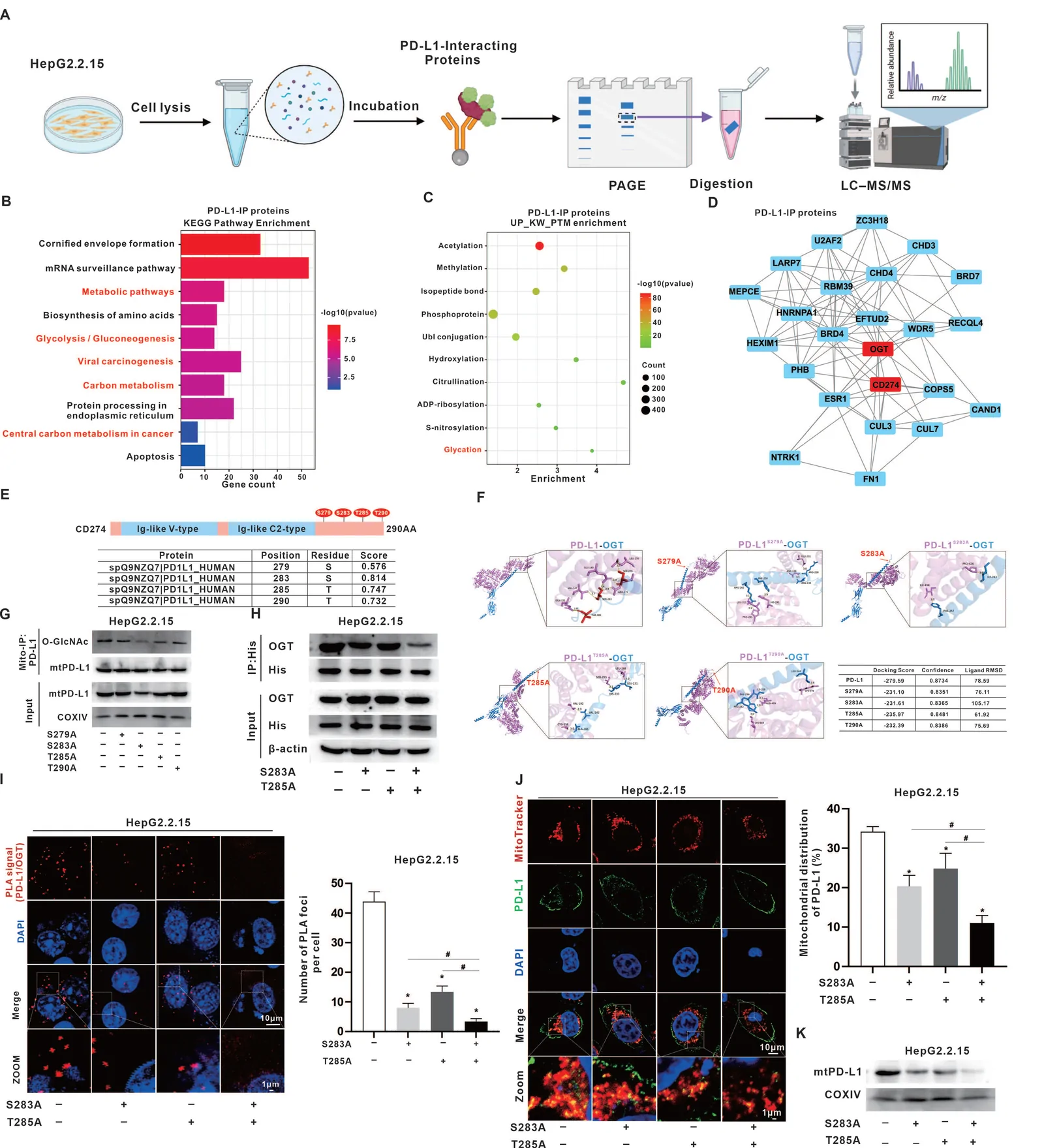
** OGT-mediated O-GlcNAcylation of PD-L1 facilitates its mitochondrial localization and promotes immune pressure-resistant phenotypes in HepG2.2.15 cells.** (A-D) PD-L1-overexpressing HepG2.2.15 cells were constructed, followed by protein extraction and subsequent co-immunoprecipitation (Co-IP) coupled with LC-MS/MS analysis (A). KEGG (B) and PTM (C) enrichment analyses were performed on the identified PD-L1-interacting proteins. (D) PPI network topology of PD-L1 Co-IP candidates, with *CD274* (PD-L1) and OGT highlighted to indicate a central PD-L1-OGT module within the interactome. (E) Schematic illustration of the domain architecture of PD-L1 and a summary table of O-GlcNAcylation sites (Ser/Thr residues), including residue positions and site-localization confidence scores. (F) *In silico* docking and modeling of the PD-L1-OGT interface for PD-L1 (wild-type, WT) and indicated alanine mutants (S279A, S283A, T285A, T290A), with corresponding docking metrics (docking score, confidence, and ligand RMSD). (G) Mitochondrial immunoprecipitation (Mito-IP) of mtPD-L1 followed by immunoblotting (IB) for O-GlcNAc to assess PD-L1 O-GlcNAcylation in mitochondria. (H) Mito-IP of His-tagged PD-L1 followed by IB for OGT to evaluate mitochondrial PD-L1-OGT association. (I) Proximity ligation assay (PLA) detecting *in situ* proximity between PD-L1 and OGT (red puncta) with nuclear counterstain (DAPI, blue) (left); quantification of PLA foci per cell under the indicated PD-L1 mutation conditions (right). Scale bar: 10 μm (main) and 1 μm (Zoom). (J) Representative immunofluorescence (IF) images showing mtPD-L1 (green) localization relative to mitochondria (MitoTracker, red). Nuclei were counterstained with DAPI (blue) (left); quantification of mitochondrial PD-L1 distribution (right). Scale bar: 10 μm (main) and 1 μm (Zoom). (K) Western blot (WB) analysis of mtPD-L1 in mitochondrial fractions. Data are mean ± SD, n = 3. *, *P* < 0.05 versus control; #, *P* < 0.05 versus the indicated group.

To further define the mechanism underlying HBx-driven O-GlcNAcylation of PD-L1, we established an integrated workflow combining PD-L1 interactome profiling, site-mapping analysis, mutagenesis, and functional validation (Fig. 3A). PD-L1 Co-IP followed by LC-MS/MS in HepG2.2.15 cells identified a PD-L1-associated protein network enriched in metabolic pathways, including glycolysis/gluconeogenesis, carbon metabolism, and central carbon metabolism in cancer, whereas PTM-enrichment analysis highlighted glycation-related signatures, collectively supporting glycosylation-dependent regulation of PD-L1 (Fig. 3B-D). Site-mapping analysis further nominated four high-confidence candidate O-GlcNAc residues within PD-L1, namely S279, S283, T285, and T290 (Fig. 3E). Structural docking indicated that alanine substitution at these residues did not substantially alter the predicted PD-L1-OGT binding score, suggesting that they are likely functional modification sites rather than major binding interfaces (Fig. 3F).

Functionally, mitochondrial pull-down assays showed that mutation of S283 or T285 markedly reduced O-GlcNAcylated mtPD-L1, whereas S279A and T290A had relatively modest effects (Fig. 3G). Consistently, exogenous pull-down and PLA assays demonstrated that either S283A or T285A weakened the PD-L1-OGT interaction, with the S283A/T285A double mutant showing the greatest reduction in PLA puncta (Fig. 3H-I). Confocal imaging, quantitative mitochondrial distribution analysis, and mitochondrial fractionation further confirmed that mutation of S283 or T285 impaired PD-L1 mitochondrial accumulation, again with the double mutant exerting the strongest inhibitory effect (Fig. 3J-K). Importantly, in PBMC co-culture assays, the S283A/T285A mutant was associated with stronger PBMC-derived cytokine release than wild-type PD-L1, as shown by increased IFN-γ and TNF-α secretion (Fig. S3A-B). This was accompanied by reduced EdU incorporation (Fig. S3C), colony formation (Fig. S3D), and migratory capacity (Fig. S3E-F), together with increased Annexin V/PI positivity (Fig. S3G), LDH release (Fig. S3H), and tumor-cell death (Fig. S3I). Collectively, these data identify S283 and T285 as major functional O-GlcNAcylation sites required for PD-L1 mitochondrial translocation and immune pressure-resistant tumor-cell phenotypes, and support a model in which HBx promotes mtPD-L1 enrichment predominantly through remodeling of the OGT/O-GlcNAc axis rather than by acting as a direct OGT scaffold.

### OSMI-1 selectively targets O-GlcNAcylation and potentiates αPD-L1-associated tumor-cell suppression *in vitro*

**Figure 4:**
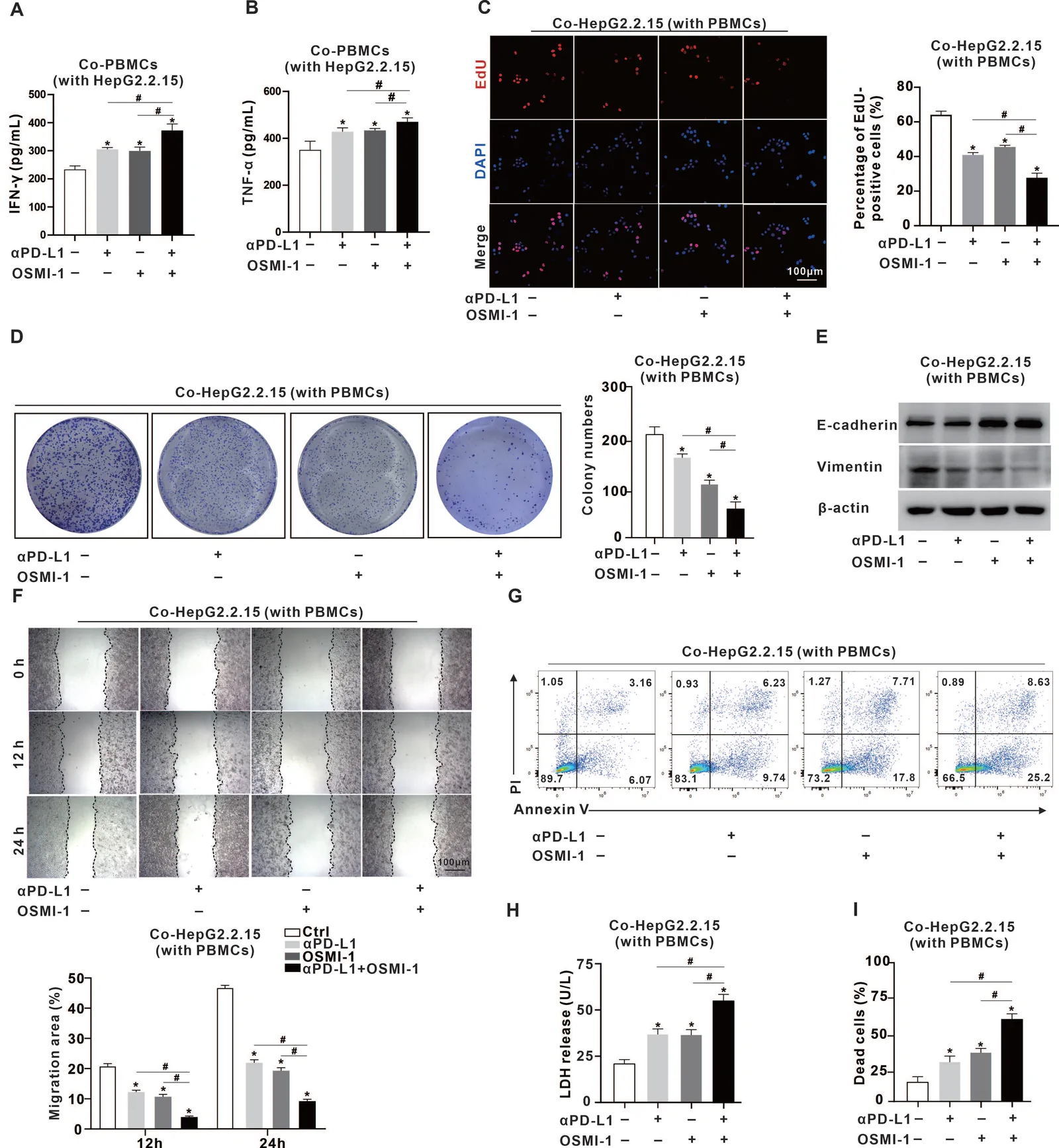
** OSMI-1 selectively targets O-GlcNAcylation and potentiates αPD-L1-associated tumor-cell suppression *in vitro*.** Activated PBMCs and HepG2.2.15 cells were co-cultured and treated with αPD-L1 (10 μg/mL), OSMI-1 (10 μM), or their combination for 24 h *in vitro*. (A-B) PBMC-derived IFN-γ (A) and TNF-α (B) levels were measured by ELISA as cytokine readouts in the PBMC-tumor co-culture system. (C-I) Tumor phenotypes were assessed in the co-cultured HepG2.2.15 cells. (C) Representative IF images of EdU incorporation assays for cell proliferation (left); the percentage of EdU-positive cells is presented in the bar graph (right); nuclei were counterstained with DAPI. Scale bar: 100 μm. (D) Representative images of colony formation assays (left); quantification of colony numbers is presented in the bar graph (right). (E) Levels of cell migration-related proteins E-cadherin and Vimentin. (F) Representative images of scratch-wound assays evaluating cell migration at 0, 12, and 24 h (upper); quantification of migration rate is presented in the bar graph (lower). (G) Representative flow cytometry plots and quantification of apoptosis by Annexin V/PI staining. (H-I) LDH release (H) and the percentage of dead cells (I) were measured as tumor-cell injury-related readouts under PBMC-associated immune pressure. Data are mean ± SD, n = 3. *, *P* < 0.05 versus control; #, *P* < 0.05 versus the indicated group.

To assess the contribution of mtPD-L1 to immunotherapy resistance, we used OSMI-1 to disrupt mtPD-L1 localization and combined it with αPD-L1 to evaluate the therapeutic impact of targeting mtPD-L1. An *in vitro* co-culture system was established using activated PBMCs and HBx-expressing HCC cells (HepG2.2.15 and MHCC-97H), followed by treatment with αPD-L1, OSMI-1, or their combination. PBMC-associated cytokine release and tumor-cell phenotypes were then assessed. Monotherapy with either αPD-L1 or OSMI-1 modestly increased PBMC-derived IFN-γ and TNF-α secretion (Fig. 4A-B and Fig. S4A-B). Notably, combined treatment produced the highest levels of both cytokines, indicating an enhanced PBMC effector-associated response in this co-culture setting.

Tumor proliferative capacity, measured by EdU incorporation and colony formation in HBx-expressing HCC cells co-cultured with PBMCs, was significantly reduced by each monotherapy and further suppressed by the combined regimen (Fig. 4C-D and Fig. S4C-D). Consistently, E-cadherin was upregulated, whereas Vimentin was downregulated (Fig. 4E and Fig. S4E). Scratch-wound assays further showed that either agent alone significantly impaired migratory capacity, with the strongest inhibition observed under combined treatment (Fig. 4F and Fig. S4F). To evaluate tumor-cell injury under PBMC-associated immune pressure, we assessed apoptosis, LDH release, and the percentage of dead cells in PBMC co-cultures. Both αPD-L1 and OSMI-1 alone increased tumor-cell injury-related readouts, whereas the combination induced the highest levels of apoptosis and LDH release (Fig. 4G-I and Fig. S4G-I).

Collectively, these findings indicate that OSMI-1-mediated blockade of O-GlcNAcylation depletes mtPD-L1 and sensitizes HBx-expressing HCC cells to αPD-L1 immunotherapy, highlighting mtPD-L1 as a key determinant of therapeutic resistance and a potential target for combination therapy.

### O-GlcNAcylation-driven mtPD-L1 promotes mitochondrial translation and rewires energy metabolism in HBx-expressing HCC

**Figure 5:**
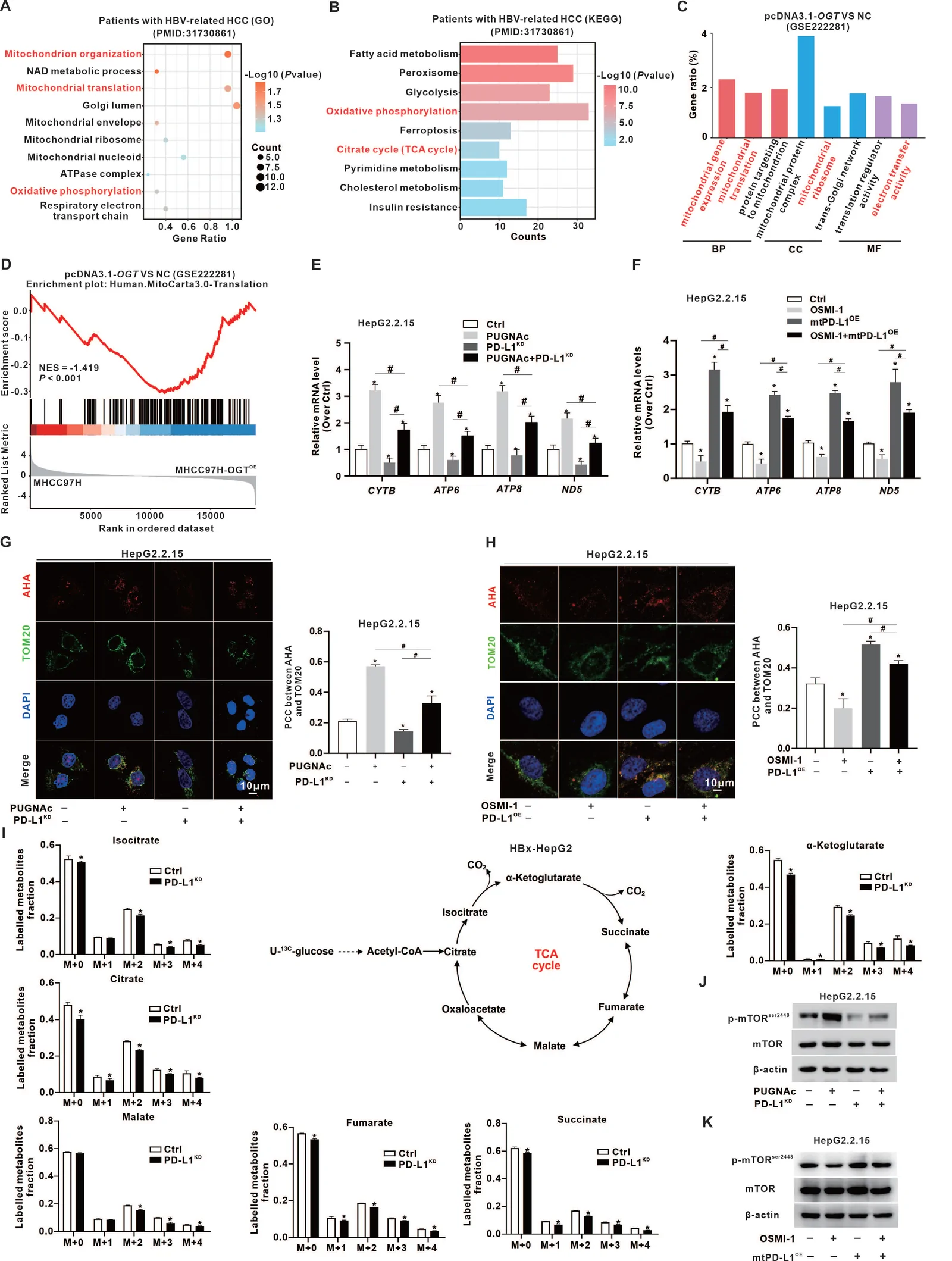
** O-GlcNAcylation-driven mtPD-L1 promotes mitochondrial translation and rewires energy metabolism in HBx-expressing HCC cells.** (A-B) GO (A) and KEGG (B) enrichment analyses of the proteomic dataset from patients with HBx-related HCC (n = 159). (C) Public GEO dataset GSE222281 was derived from OGT-high-expressing MHCC-97H cells transfected with pcDNA3.1-*OGT* (n = 4) versus vector control (n = 4). GO enrichment of DEGs is shown. (D) GSEA comparing OGT-high-expressing MHCC-97H cells with vector-control MHCC-97H cells. (E-F) HepG2.2.15 cells with CRISPR/Cas9-mediated PD-L1 knockdown (PD-L1^KD^) were treated with or without PUGNAc (10 μM) (E) or OSMI-1 (10 μM) (F). Relative mRNA levels of mitochondrial-encoded genes were quantified by qRT-PCR after 12 h of treatment. (G-H) Representative IF images of AHA-labeled nascent mitochondrial proteins (fluorescence intensity) after 24 h treatment. Nuclei were counterstained with DAPI (left); quantification of AHA-TOM20 colocalization using Pearson's correlation coefficient (PCC) is presented in the bar graph (right). Scale bar: 10 μm. (I) Stable-isotope tracing with [U-^13^C] glucose was used to quantify the labeled metabolite fractions of TCA-cycle intermediates in PD-L1^KD^ HepG2.2.15 cells. (J-K) Levels of phosphorylated mTOR (p-mTOR^Ser2448^) and total mTOR were detected by WB. Data are mean ± SD, n = 3. *, *P* < 0.05 versus control; #, *P* < 0.05 versus the indicated group.

To investigate the functional consequences of O-GlcNAcylation-driven mtPD-L1 localization, we integrated proteomic and transcriptomic data from HBx-related HCC. GO analysis of the HBx-positive tumor proteome showed significant enrichment of "mitochondrion organization", "mitochondrial translation", "oxidative phosphorylation", and "TCA cycle" pathways (Fig. 5A-B). Consistently, transcriptomic analysis of OGT-high MHCC-97H cells revealed enrichment of "mitochondrial gene expression", "mitochondrial translation", "mitochondrial ribosome", and "electron transfer activity" (Fig. 5C). Moreover, GSEA of the dataset GSE222281 demonstrated significant suppression of the mitochondrial translation signature in *OGT*-high versus* OGT*-low tumors (Fig. 5D), further supporting the clinical relevance of this finding.

To establish causality and minimize potential cell line-specific pharmacologic bias, we combined genetic and pharmacologic perturbations to interrogate mtPD-L1 and its O-GlcNAcylation status in two HBx-positive HCC models. In HepG2.2.15 and MHCC-97H cells, treatment with the OGA inhibitor PUGNAc increased mitochondria-encoded transcripts (including *CYTB*, *ATP6*, *ATP8*, and *ND5*), whereas PD-L1 knockdown reversed these increases (Fig. 5E-F and Fig. S5A-B). *De novo* mitochondrial translation, assessed by AHA labeling coupled with click chemistry 28, was similarly stimulated by PUGNAc and attenuated by PD-L1 depletion (Fig. 5G-H and Fig. S5C-D). Conversely, OSMI-1 treatment persistently suppressed mitochondrial transcription and bioenergetic readouts, whereas mtPD-L1 overexpression markedly attenuated these effects, indicating that the response was primarily PD-L1-dependent rather than a compound-specific artifact. Consistently, CRISPR/Cas9-mediated PD-L1 knockdown in HepG2.2.15 cells reduced both mitochondrial gene transcription and AHA fluorescence intensity (Fig. 5E-H and Fig. S5A-D).

Because mitochondrial translation generates essential subunits of the respiratory chain, we next examined its metabolic consequences. Stable-isotope tracing with [U-^13^C] glucose showed that PD-L1 depletion in HepG2.2.15 cells significantly decreased the M+2 isotopologues of TCA-cycle intermediates (citrate, isocitrate, α-ketoglutarate, succinate, fumarate, and malate), indicating impaired TCA-cycle flux (Fig. 5I). In both HepG2.2.15 and MHCC-97H cells, mtPD-L1 overexpression or PUGNAc treatment increased the NAD^+^/NADH ratio (Fig. S5E-F), ATP production (Fig. S5G-H), and mTOR activation (Fig. 5J-K), whereas OSMI-1 treatment or PD-L1 depletion induced bioenergetic collapse and reduced p-mTOR^Ser2448^ levels (Fig. 5J-K). Collectively, these cross-inhibitor and cross-cell-line data demonstrate that O-GlcNAcylation-dependent mtPD-L1 sustains mitochondrial translation, preserves TCA-cycle activity and bioenergetic output, and activates mTOR signaling, thereby reprogramming mitochondrial metabolism to support immunotherapeutic resistance in HBx-positive HCC.

### mtPD-L1 drives mitochondrial biogenesis via the mTOR/PGC-1α axis in HBx-expressing HCC cells

GO analysis of proteomic data from HBV-related HCC tissues revealed significant enrichment of "mitochondrial organization" (Fig. 5A), suggesting enhanced mitochondrial biogenesis in this subtype. Reanalysis of the TCGA-LIHC cohort further showed that transcripts encoding mitochondrial biogenesis regulators *CHCHD2* and *NFE2L2* were markedly upregulated in tumors compared with adjacent tissues (Fig. S6A). Kaplan-Meier analysis demonstrated that high expression of either gene was independently associated with shorter overall survival (OS) (Fig. S6B), highlighting the critical role of mitochondrial biogenesis in HBV-related HCC.

We therefore hypothesized that enhanced mitochondrial translation increases the production of mtDNA-encoded respiratory subunits while activating the energy sensor mTOR, thereby reprogramming nuclear gene expression 32. Consistent with this model, reanalysis of the TCGA-LIHC cohort revealed a strong positive correlation between *CD274* and mitochondrial biogenesis-related transcripts in HBV-related HCC (Fig. S6C). IHC further showed that mitochondrial markers PGC-1α, NRF1, and TFAM were expressed at significantly higher levels in HBV-positive than in HBV-negative HCC tissues, levels that closely paralleled PD-L1 abundance (Fig. S6D).

**Figure 6:**
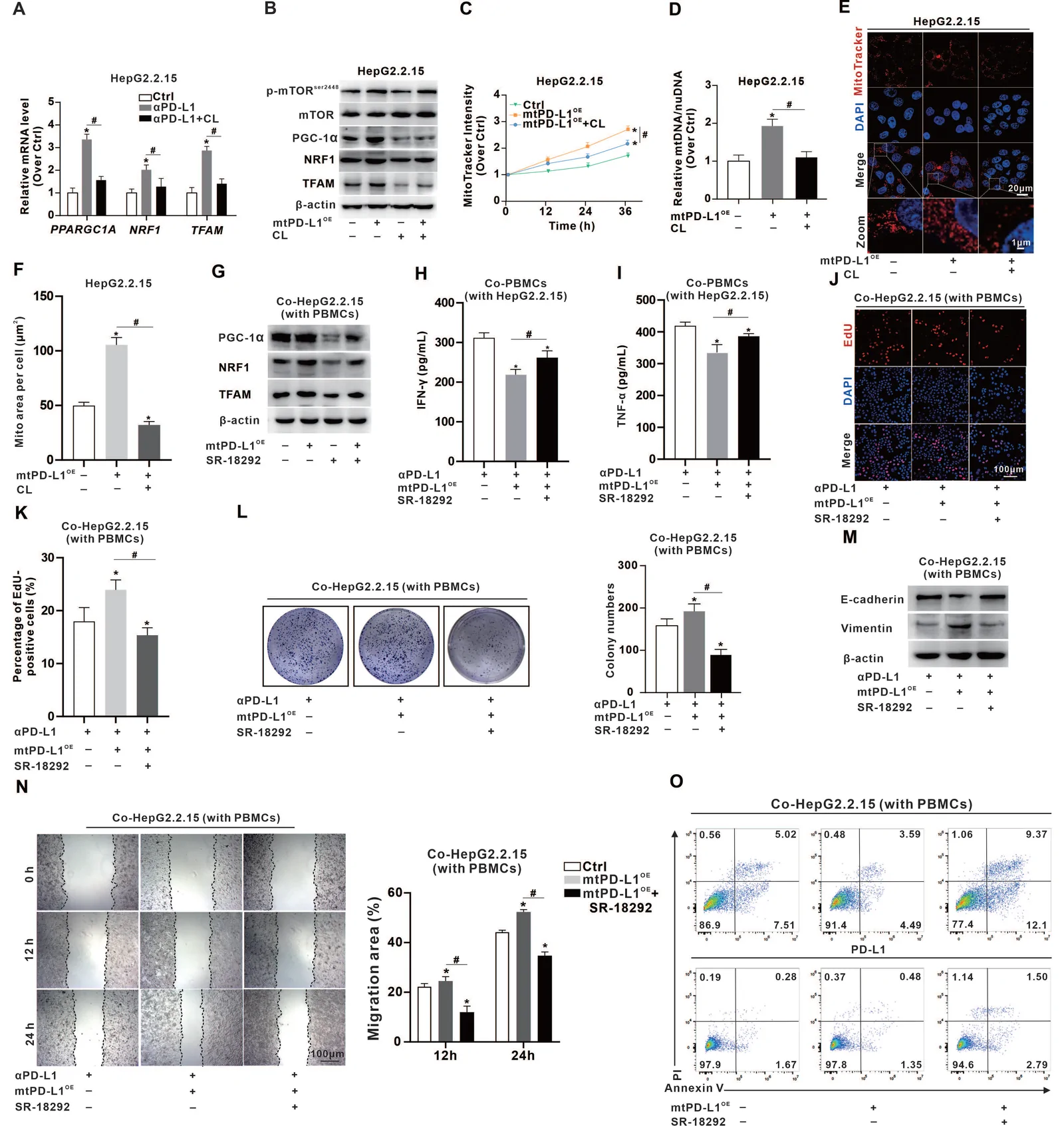
** mtPD-L1 drives mitochondrial biogenesis via the mTOR/PGC-1α axis in HBx-expressing HCC cells.** (A-F) mtPD-L1^OE^ HepG2.2.15 cells were treated with or without chloramphenicol (CL, 100 μg/mL), a selective inhibitor of mitochondrial translation. (A) mRNA levels of mitochondrial biogenesis-related genes in cells treated for 12 h were quantified by qRT-PCR. (B) Levels of p-mTOR^Ser2448^, total mTOR, and mitochondrial biogenesis-related PGC-1α, NRF1, and TFAM proteins in cells treated for 24 h were detected by WB. (C) Quantification of mitochondrial biogenesis by flow cytometry analysis of MitoTracker fluorescence intensity in cells treated for 12, 24, and 36 h. (D) Quantification of mtDNA copy numbers in cells treated for 24 h, with nDNA copy numbers used as an internal reference. (E) Representative IF images of MitoTracker fluorescence intensity in cells treated for 24 h; nuclei were counterstained with DAPI. Scale bar: 20 μm (main) and 1 μm (Zoom). (F) Quantification of MitoTracker fluorescence intensity. (G-O) mtPD-L1^OE^ HepG2.2.15 cells were co-cultured with activated PBMCs and treated with or without αPD-L1 (10 μg/mL) and/or SR-18292 (10 μM) for 24 h. (G) Levels of PGC-1α, NRF1, and TFAM proteins in HepG2.2.15 cells treated for 24 h were detected by WB. (H-I) PBMC-derived IFN-γ (H) and TNF-α (I) levels were measured by ELISA. (J-O) Survival metrics of co-cultured mtPD-L1^OE^ HepG2.2.15 cells. (J) Representative images of EdU incorporation assays. Scale bar: 100 μm. (K) Quantification of EdU-positive cells is shown in the bar graph. (L) Representative images of colony formation assays (left); quantification of colony numbers is presented in the bar graph (right). (M) Levels of cell migration-related proteins E-cadherin and Vimentin. (N) Representative images of scratch-wound assays evaluating cell migration at 0, 12, and 24 h (left); quantification of migration rate is presented in the bar graph (right). (O) Representative flow cytometry plots and quantification of apoptosis by Annexin V/PI staining. Data are mean ± SD, n = 3. *, *P* < 0.05 versus control; #, *P* < 0.05 versus the indicated group.

TEM of mtPD-L1^OE^ MHCC-97H cells revealed enlarged mitochondria with dense cristae and increased fusion (Fig. S6E), consistent with enhanced mitochondrial biogenesis. Concordantly, transcript levels of *PPARGC1A* (encoding PGC-1α), *NRF1*, and *TFAM* were upregulated (Fig. 6A and Fig. S6F), accompanied by increased p- mTOR^Ser2448^, PGC-1α, NRF1, and TFAM protein levels in both mtPD-L1^OE^ MHCC-97H and HepG2.2.15 cells (Fig. 6B and Fig. S6G). These effects were abolished by chloramphenicol (CL), indicating that *de novo* synthesis of mtDNA-encoded proteins is required for the biogenic response. Real-time MitoTracker flow cytometry showed a rapid increase in mitochondrial mass within ~36 h in mtPD-L1-overexpressing cells, which was blunted by CL (Fig. 6C and Fig. S6H). Consistently, mtDNA copy number and per-cell MitoTracker intensity were increased by mtPD-L1 overexpression but reduced by CL (Fig. 6D-F and Fig. S6I-K). To establish causality of the mTOR-PGC-1α axis, we pharmacologically modulated mTOR activity. Rapamycin (RAPA) reversed the mitochondrial biogenesis program in mtPD-L1^OE^ MHCC-97H cells, whereas the mTOR activator MHY1485 rescued these markers in PD-L1^KD^ HepG2.2.15 cells (Fig. S6L-P). Collectively, these findings demonstrate that mtPD-L1 promotes mitochondrial biogenesis in HBx-expressing HCC cells through mTOR-dependent induction of PGC-1α.

To determine whether mtPD-L1-driven mitochondrial biogenesis constrains immunotherapeutic efficacy, we pretreated HBx-expressing HCC cells with SR-18292, a selective inhibitor of PGC-1α-dependent mitochondrial biogenesis, replaced the medium, and then co-cultured the cells with PBMCs in the presence or absence of αPD-L1, thereby restricting SR-18292 exposure to tumor cells. Under these conditions, SR-18292 reduced PGC-1α, NRF1, and TFAM expression in both mtPD-L1^OE^ MHCC-97H and HepG2.2.15 cells (Fig. 6G and Fig. S7A). Notably, SR-18292 enhanced αPD-L1-associated IFN-γ and TNF-α release in the PBMC co-culture system (Fig. 6H-I and Fig. S7B-C). Functionally, combined SR-18292 and αPD-L1 treatment markedly suppressed EdU incorporation (Fig. 6J-K and Fig. S7D), colony formation (Fig. 6L and Fig. S7E), and increased E-cadherin expression while decreasing Vimentin expression (Fig. 6M and Fig. S7F), suppressed wound closure (Fig. 6N and Fig. S7G-H), and increased apoptosis (Fig. 6O and Fig. S7I), LDH release (Fig. S7J), and tumor-cell death (Fig. S7K) in HBx-expressing HCC cells. Collectively, these findings identify mtPD-L1-driven, mTOR-PGC-1α-dependent mitochondrial biogenesis as a metabolic checkpoint that supports tumor-cell fitness under αPD-L1-associated immune pressure.

### OSMI-1 synergizes with αPD-L1 to sensitize HBx-Hepa1-6 cells to soluble effector factors released by anti-CD3/CD28-preactivated CD8⁺ T-cells* in vitro*

To further examine whether OSMI-1 alters the response of HBx-related tumor cells to CD8⁺ T-cell-associated soluble effector pressure in a murine system, we used purified CD8⁺ T-cells from OT-1 mice as a standardized source of murine CD8⁺ T-cells for the non-contact soluble-factor response assay. The central focus of this study is the tumor-cell-intrinsic role of HBx-driven mitochondrial PD-L1, particularly how O-GlcNAcylation of mtPD-L1 reshapes mitochondrial metabolic fitness and thereby influences the susceptibility of HBx-related tumor cells to immune effector stress. The combination of OSMI-1 with αPD-L1 was designed to determine whether pharmacological inhibition of PD-L1 O-GlcNAcylation could enhance the tumor-cell response to immune effector pressure by weakening this tumor-cell-intrinsic resistance mechanism. In the preceding experiments, PBMC-based co-culture systems were primarily used to assess immune effector-associated tumor-cell responses. The OT-1-derived CD8⁺ T-cell non-contact soluble-factor response assay was included as a complementary T-cell-focused assay in a murine setting, using a defined CD8⁺ T-cell source. We recognize that OT-1 CD8⁺ T-cells classically recognize the OVA-derived SIINFEKL peptide presented by H-2Kᵇ. However, HBx-Hepa1-6 cells used in this study do not express OVA, and neither OVA protein nor SIINFEKL peptide was added to the co-culture system. Moreover, OT-1-derived CD8⁺ T-cells were first preactivated *ex vivo* with anti-CD3/CD28 magnetic beads and then placed in the upper chamber of a non-contact Transwell system, whereas HBx-Hepa1-6 cells were seeded in the lower chamber. Therefore, direct T-cell-tumor-cell contact, immunological synapse formation, contact-dependent cytotoxicity, and SIINFEKL-H-2Kᵇ-restricted OT-1 TCR-mediated tumor recognition could not be assessed in this assay.

**Figure 7:**
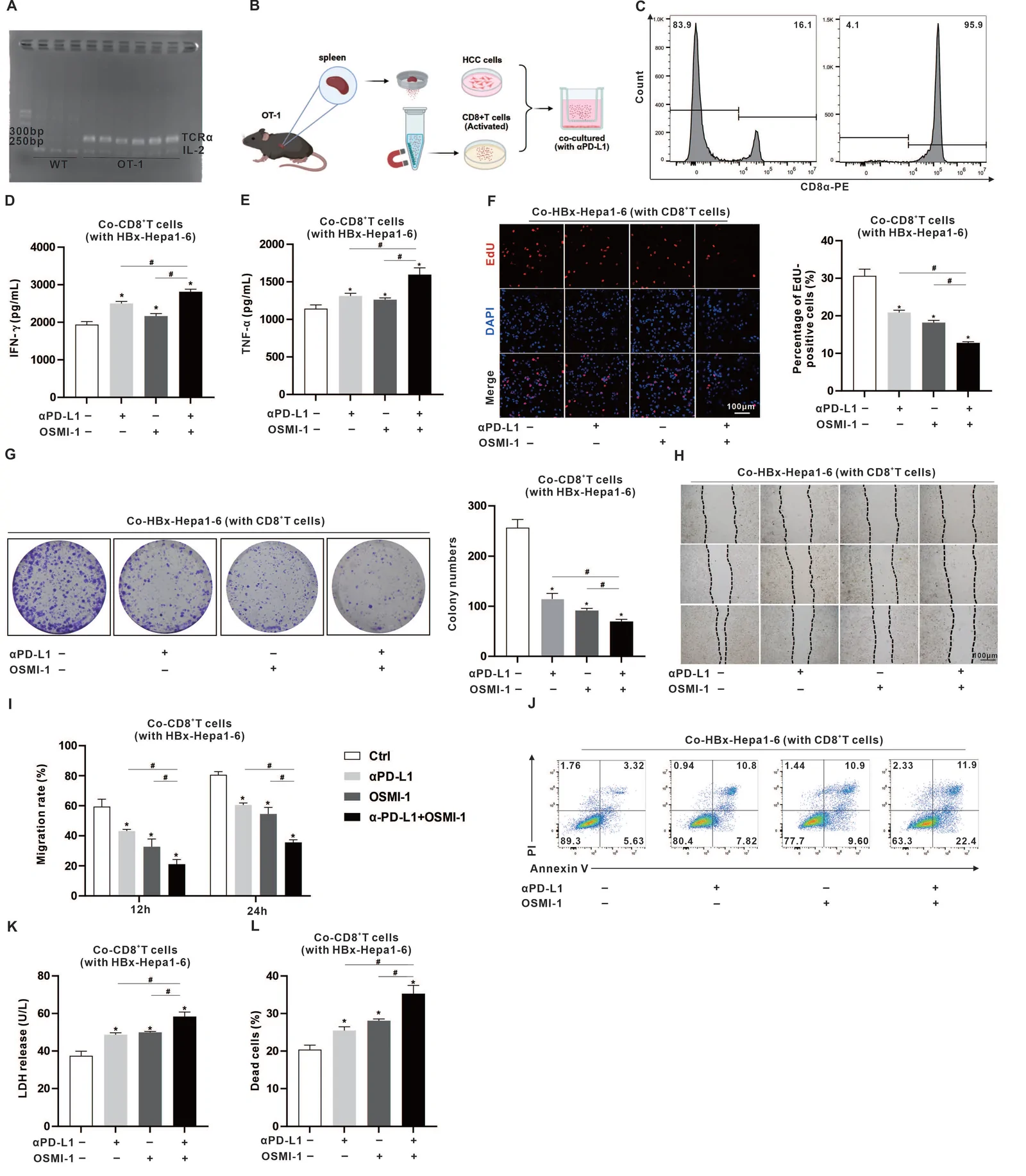
** OSMI-1 synergizes with αPD-L1 to sensitize HBx-Hepa1-6 cells to soluble effector factors released by anti-CD3/CD28-preactivated CD8⁺ T-cells *in vitro*.** (A) Representative PCR genotyping of OT-1 mice using primers for TCRα and IL-2. (B) Schematic illustration of the experimental workflow: splenic CD8⁺ T-cells isolated from OT-1 mice were preactivated *ex vivo* with anti-CD3/CD28 magnetic beads and co-cultured with HBx-Hepa1-6 cells in a non-contact Transwell soluble-factor response system in the presence or absence of αPD-L1 and/or OSMI-1 (10 μM, 24 h). No OVA protein or SIINFEKL peptide was added. Thus, this assay was designed to assess HBx-Hepa1-6 cell responses to soluble effector mediators released by anti-CD3/CD28-preactivated CD8⁺ T-cells rather than SIINFEKL-H-2Kᵇ-restricted OT-1 TCR-mediated tumor recognition. (C) Flow-cytometric assessment of CD8⁺ T-cell purity (CD8α-PE) before and after isolation. (D-L) Anti-CD3/CD28-preactivated OT-1-derived CD8⁺ T-cells and HBx-Hepa1-6 cells were co-cultured in the non-contact Transwell soluble-factor response system and treated with αPD-L1 (10 μg/mL), OSMI-1 (10 μM), or their combination for 24 h *in vitro*. (D-E) IFN-γ (D) and TNF-α (E) levels in the culture supernatant were measured by ELISA and are presented as cytokine levels in the co-culture supernatant from the non-contact Transwell soluble-factor response system, rather than as evidence of antigen-specific OT-1 TCR-mediated recognition or T-cell activation against HBx-Hepa1-6 cells. (F) Representative IF images of EdU incorporation assays showing cell proliferation (left); quantification of the percentage of EdU-positive cells is shown in the bar graph (right); nuclei were counterstained with DAPI. Scale bar: 100 μm. (G) Representative images of colony formation assays (left); quantification of colony numbers is shown in the bar graph (right). (H) Representative images of scratch-wound assays evaluating cell migration at 0, 12, and 24 h. (I) Quantification of migration rate. (J) Representative flow cytometry plots and quantification of apoptosis by Annexin V/PI staining. (K-L) LDH release (K) and percentage of dead cells (L) were measured as tumor-cell injury-related readouts in the non-contact Transwell soluble-factor response system. Data are mean ± SD, n = 3. *, *P* < 0.05 versus control; #, *P* < 0.05 versus the indicated group.

OT-1 identity was confirmed by genotyping, and splenic CD8⁺ T-cells were isolated at high purity and subsequently activated, with > 95% CD8α⁺ cells detected by flow cytometry (Fig. 7A-C). Under this non-contact soluble-factor response condition, IFN-γ and TNF-α levels in the co-culture supernatant were increased by αPD-L1 or OSMI-1 alone and were maximally elevated by the combination treatment (Fig. 7D-E). Consistent with increased tumor-cell vulnerability to an anti-CD3/CD28-preactivated CD8⁺ T-cell-associated soluble effector environment, combined treatment with αPD-L1 and OSMI-1 more effectively suppressed HBx-Hepa1-6 cell proliferation, clonogenic growth, and migration than either monotherapy, as shown by EdU incorporation (Fig. 7F), colony formation (Fig. 7G), and scratch-wound assays (Fig. 7H-I). Moreover, the combination regimen produced the strongest tumor-cell injury, evidenced by increased Annexin V/PI-positive cells (Fig. 7J), elevated LDH release (Fig. 7K), and a higher dead-cell fraction (Fig. 7L). These findings should therefore be interpreted as evidence that OSMI-1, particularly in combination with αPD-L1, increases the susceptibility of HBx-Hepa1-6 cells to soluble effector factors released by anti-CD3/CD28-preactivated CD8⁺ T-cells, rather than as evidence of SIINFEKL-H-2Kᵇ-restricted OT-1 TCR-mediated tumor killing. Mechanistically, this conservative interpretation is consistent with our central model that OSMI-1 disrupts the O-GlcNAc-mtPD-L1-mTOR/PGC-1α axis and weakens the metabolic fitness of HBx-related tumor cells, thereby impairing mitochondrial metabolic fitness and rendering tumor cells more vulnerable to soluble immune effector-associated stress. Thus, the OT-1-derived CD8⁺ T-cell non-contact soluble-factor response assay provides complementary support for a tumor-cell-centered mechanism of sensitization, rather than direct evidence of antigen-specific OT-1 TCR-mediated tumor-cell killing.

To exclude a confounding direct effect of OSMI-1 on CD8⁺ T-cells, we further performed T-cell-only assays using purified CD8⁺ T-cells. Under the concentrations used, OSMI-1 did not affect T-cell viability or alter the CD8α profile (Fig. S8A-B), indicating no overt toxicity or perturbation of CD8⁺ T-cell identity. Importantly, OSMI-1 did not suppress cytokine levels; IFN-γ and TNF-α concentrations were maintained or modestly increased (Fig. S8C-D), with the highest cytokine levels observed in the OSMI-1 plus αPD-L1 group. Together, these data support a conservative conclusion that OSMI-1 cooperates with αPD-L1 to sensitize HBx-Hepa1-6 cells to a soluble effector environment generated by anti-CD3/CD28-preactivated CD8⁺ T-cells, although this assay does not demonstrate antigen-specific OT-1 TCR-mediated tumor-cell killing.

### OSMI-1 synergizes with αPD-L1 to overcome mtPD-L1-mediated resistance in HBx-driven HCC *in vivo*

**Figure 8:**
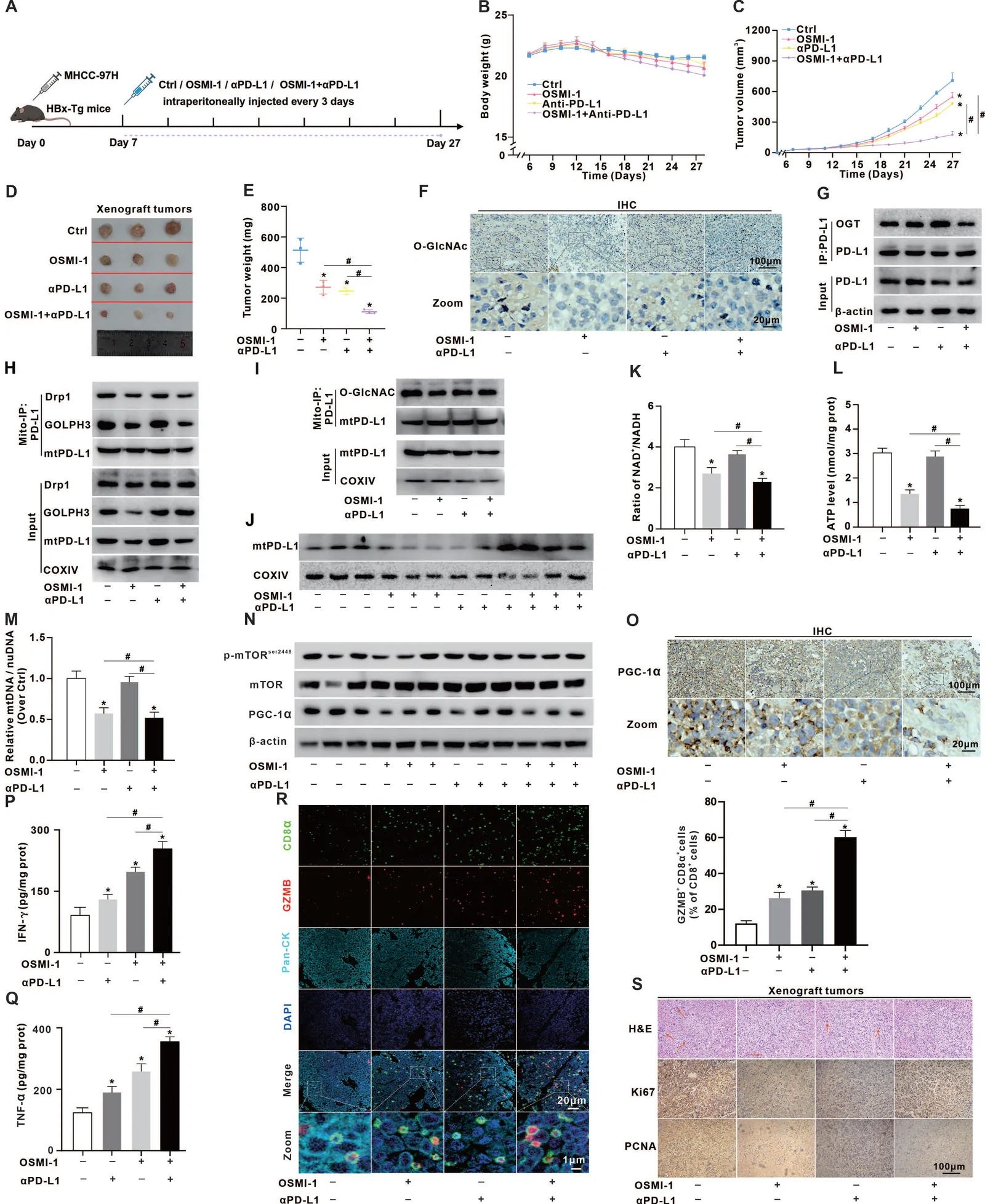
** OSMI-1 synergizes with αPD-L1 to overcome mtPD-L1-mediated resistance in HBV-driven HCC *in vivo*.** (A) Schematic timeline of subcutaneous MHCC-97H xenograft establishment in HBx-Tg mice and randomized treatment with vehicle (Ctrl), OSMI-1 (10 mg/kg, i.p.), αPD-L1 (5 mg/kg, i.p.), or combination therapy administered by intraperitoneal injection every 3 days for 27 days. n = 5 per group. (B) Body weight curves of HBx-Tg mice monitored throughout the treatment period. (C) Xenograft tumor volume measured every third day; tumor growth curves depict changes over 27 days. (D) Representative photographs of xenograft tumors at study endpoint. (E) Endpoint tumor weight in all groups. (F) Representative IHC staining of global O-GlcNAc in tumor sections. Scale bar: 100 μm (main) and 20 μm (Zoom). (G) Co-IP assessing PD-L1 interaction with OGT in tumor lysates. (H) Mito-IP assessing mtPD-L1 interaction with the GOLPH3/Drp1 complex in mitochondrial fractions from tumors. (I) Mito-IP assessing O-GlcNAcylation of mtPD-L1 in mitochondrial fractions from tumors. (J) Levels of mtPD-L1 in mitochondrial fractions from tumors were detected by WB. (K-L) Quantification of NAD^+^/NADH ratio (K) and ATP levels (L) in xenograft tumors to evaluate mitochondrial metabolic flux. (M) mtDNA copy numbers in tumors were quantified by qRT-PCR, with nDNA copy numbers used as an internal reference. (N) Levels of p-mTOR^Ser2448^, total mTOR, and mitochondrial biogenesis-related PGC-1α proteins in tumor lysates were detected by WB. (O) Representative IHC staining of PGC-1α in tumor sections. Scale bar: 100 μm (main) and 20 μm (Zoom). (P-Q) Quantification of IFN-γ (P) and TNF-α (Q) in tumor homogenates was performed to assess local cytokine responses. (R) Representative multiplex immunofluorescence images of tumor sections stained for CD8α (green), GZMB (granzyme B; red), and Pan-CK (cyan) with nuclear counterstain DAPI (blue) (left); quantification of CD8α⁺GZMB⁺ cells as a percentage of total CD8α⁺ cells is shown in the bar graph (right). Scale bar: 20 μm (main) and 1 μm (Zoom). (S) Representative images of H&E staining and IHC staining of Ki67 and PCNA in tumor sections. Scale bar: 100 μm. Data are mean ± SD, n = 3. *, *P* < 0.05 versus control; #, *P* < 0.05 versus the indicated group.

To extend our *in vitro* findings on mtPD-L1-mediated resistance to checkpoint blockade into an immunocompetent *in vivo* setting, we conducted two independent subcutaneous tumor experiments in HBx-Tg mice. In the first, a CD8-depleted MHCC-97H xenograft model was used to evaluate the tumor growth-suppressive effect in a human HCC setting. In the second, an immunocompetent syngeneic model was established by implanting Hepa1-6 cells to determine whether mtPD-L1 targeting could sensitize tumors to immune checkpoint blockade in an intact host immune system. In both models, treatment with either OSMI-1 or αPD-L1 alone significantly reduced tumor growth and endpoint tumor weight relative to the vehicle control, whereas the combination treatment produced the strongest tumor suppression (Fig. 8A-E and Fig. S9A-D). Notably, the superior efficacy observed in the Hepa1-6 syngeneic model supports the involvement of host immune effector mechanisms, in addition to tumor-cell-intrinsic growth suppression.

We next investigated whether OSMI-1 disrupts the Golgi-mitochondria trafficking of mtPD-L1 within the tumor microenvironment. IHC showed a global reduction in protein O-GlcNAcylation in xenograft tumors from the OSMI-1 and combination groups (Fig. 8F and Fig. S9E). Co-IP of tumor lysates showed that OSMI-1 markedly weakened the interaction between PD-L1 and OGT (Fig. 8G), as well as the association of PD-L1 with the GOLPH3/Drp1 mitochondrial complex in Mito-IP assays (Fig. 8H). Consistently, Mito-IP further demonstrated that OSMI-1 reduced O-GlcNAcylation of mtPD-L1 (Fig. 8I) and concomitantly decreased its mitochondrial localization (Fig. 8J), indicating that OSMI-1 suppresses mtPD-L1 trafficking by blocking its O-GlcNAc-dependent Golgi-mitochondria shuttle in HBV-related HCC *in vivo*. Functionally, OSMI-1 disrupted mitochondrial energy homeostasis in xenograft tumors from HBx-Tg mice, as evidenced by reduced NAD⁺/NADH ratios and ATP content (Fig. 8K-L), decreased mtDNA copy number (Fig. 8M), and downregulation of p-mTOR^Ser2448^ and PGC-1α (Fig. 8N-O and Fig. S9F). Together, these findings demonstrate that O-GlcNAcylation of mtPD-L1 is required to sustain mitochondrial biogenesis and bioenergetics in HBx-positive HCC tumors.

At the tumor-immune interface, either OSMI-1 or αPD-L1 alone increased intratumoral IFN-γ and TNF-α levels, whereas the combination elicited the strongest cytokine response (Fig. 8P-Q). To more accurately assess CD8⁺ T-cell infiltration and granzyme B-associated effector status in tumor tissues, we performed multiplex immunofluorescence staining using CD8α, GZMB, Pan-CK, and DAPI. Pan-CK was used to delineate epithelial tumor cells, whereas CD8α was used to identify infiltrating T cells. The staining showed that CD8α-positive cells were present as discrete infiltrating immune cells and were clearly distinguishable from Pan-CK-positive tumor cells, excluding broad CD8α expression by tumor cells (Fig. 8R). We further quantified the proportion of GZMB⁺CD8α⁺ cells among CD8α⁺ cells. OSMI-1 or αPD-L1 alone increased this proportion, and the combination treatment produced the most pronounced increase (Fig. 8R). These results support an increased granzyme B-associated effector phenotype among infiltrating CD8α⁺ T-cells after combined OSMI-1 and αPD-L1 treatment.

Consistently, increased E-cadherin and reduced Vimentin expression indicated impaired migratory potential of tumor cells (Fig. S9G). H&E staining revealed restoration of nuclear polarity and fewer mitotic figures in all treatment groups, most prominently in the combination arm, and IHC further confirmed significant suppression of Ki67 and PCNA, with the lowest proliferation indices observed after combined treatment (Fig. 8S and Fig. S9H). To assess clinical relevance, we next analyzed an exploratory cohort of ICB-treated HBV-HCC specimens (n = 10 pairs of tumor and adjacent liver tissues). IHC demonstrated that PD-L1, global O-GlcNAcylation, PGC-1α, and mTOR were all significantly elevated in tumors relative to adjacent tissues (Fig. S10A-B). Moreover, within tumor samples, PD-L1 expression positively correlated with global O-GlcNAcylation (*R* = 0.77, *P* = 0.0085), PGC-1α (*R* = 0.65, *P* = 0.042), and mTOR (*R* = 0.59, *P* = 0.02), supporting a clinical association between PD-L1 upregulation, enhanced O-GlcNAc signaling, and activation of a mitochondrial biogenesis-related program (Fig. S10C). Collectively, these *in vivo* and patient-based data indicate that OSMI-1 blocks O-GlcNAcylation of PD-L1 and its mitochondrial localization, thereby suppressing mitochondrial biogenesis and metabolic fitness and enhancing the responsiveness of HBx-driven HCC to αPD-L1 therapy.

## Discussion

O-GlcNAcylation is frequently upregulated in HCC, where it drives hepatocarcinogenesis by promoting cell-cycle progression, DNA damage repair, and pro-proliferative signaling 33-35. Here, we show that HBx-driven O-GlcNAcylation confers both proliferative and metabolic advantages on HBx-expressing HCC cells, as evidenced by increased EdU incorporation and clonogenic growth, both of which were reversed by the selective OGT inhibitor OSMI-1. *In vivo*, OSMI-1 monotherapy reduced HCC xenograft tumor burden, depleted ATP and NAD^+^ pools, and suppressed mitochondrial biogenesis programs, identifying O-GlcNAcylation as a central metabolic driver of HBx-expressing HCC.

An unexpected finding is the functional coupling between the Golgi apparatus and mitochondria mediated by O-GlcNAcylated PD-L1. Although Golgi-mitochondria membrane contact sites (MCSs) are known to facilitate trafficking of lipids and proteins 30, 36, 37, their role in immune checkpoint regulation has remained unclear. Here, we identify a PI(4)P-dependent trafficking pathway involving the Golgi protein GOLPH3 and the mitochondrial fission regulator Drp1 that mediates the OGT-dependent delivery of O-GlcNAcylated PD-L1 to mitochondria. Mutation of the PI(4)P-binding residue in GOLPH3 (R90L) selectively abolished mtPD-L1 localization, indicating that both O-GlcNAcylation and intact Golgi-mitochondria communication are required for this process. Mechanistically, our data support a model in which the GOLPH3-Drp1 complex primarily functions as a spatial organizing and docking platform at the Golgi-mitochondria interface, coupling mtPD-L1 trafficking to downstream mTOR/PGC-1α signaling, rather than acting mainly through canonical Drp1-dependent mitochondrial fission. Consistent with this interpretation, we did not observe prominent mitochondrial fragmentation or other typical features of enhanced fission in our ultrastructural and imaging analyses. Instead, upon mitochondrial translocation, mtPD-L1 drives mTOR/PGC-1α-dependent mitochondrial biogenesis, respiratory metabolism, and resistance to immune effector-associated stress in HBx-expressing HCC cells. From a therapeutic perspective, targeting O-GlcNAcylation with OSMI-1 disrupts the association of PD-L1 with the GOLPH3-Drp1 complex, depletes mtPD-L1, impairs mitochondrial energetics, and sensitizes HBx-expressing HCC to αPD-L1 therapy. Given that OSMI-1 globally inhibits OGT, however, its effects are likely mediated predominantly, rather than exclusively, through the O-GlcNAc-mtPD-L1-mTOR/PGC-1α axis. In line with this view, exogenous mtPD-L1 remained able to activate mTOR/PGC-1α under OGT inhibition, whereas PD-L1 depletion impaired activation of this pathway even when global O-GlcNAcylation was enhanced, supporting mtPD-L1 as a major regulatory node while also implying additional contributions from other O-GlcNAc-regulated proteins, including components of the GOLPH3/mTORC1 network 38, 39. Collectively, our study reveals a post-translational mechanism governing mtPD-L1 localization and identifies a Golgi-mitochondria signaling axis that links mtPD-L1 to therapeutic resistance under immune pressure, while suggesting that contributory roles of parallel O-GlcNAc-regulated pathways cannot be fully excluded.

This study establishes mtPD-L1 as a metabolic-immune hub in HBx-driven HCC. HBx-driven mtPD-L1 import is governed by OGT-mediated O-GlcNAcylation and Golgi-mitochondria communication. Consistent with previous reports that OGT expression and global O-GlcNAcylation are elevated in HBV-related HCC and facilitate viral adaptation 40, 41, our data support a model in which HBx enhances PD-L1 O-GlcNAcylation through indirect remodeling of the OGT-O-GlcNAc axis, likely via upstream transcriptional and metabolic programs, rather than by functioning as a dedicated scaffold for direct HBx-OGT coupling. In contrast to membrane-bound PD-L1 (mPD-L1), which is readily neutralized by αPD-L1, mtPD-L1 remains inaccessible to antibody-based targeting and drives non-immune functions that support tumor growth and therapy resistance 42. Mechanistically, we demonstrate that mtPD-L1 enhances mitochondrial translation and biogenesis, thereby increasing ATP and NAD^+^ pools required for HBx-expressing HCC cell proliferation. In HBx-Tg tumor-bearing mice and HBx-expressing HCC cell lines, high mtPD-L1 levels were accompanied by increased proliferative markers, enhanced tumor heterogeneity, elevated expression of mitochondria-encoded respiratory complex transcripts, intensified Click-iT AHA labeling of mitochondrial translation products, and accumulation of TCA-cycle metabolites. Conversely, depletion of mtPD-L1 impaired mitochondrial protein synthesis, reduced ATP and NAD⁺ levels, and suppressed HBx-expressing HCC cell growth. These findings align with emerging evidence that enhanced mitochondrial biogenesis and translation contribute to therapy resistance and immune pressure adaptation 43, 44. In HCC, heightened mitochondrial biogenesis can drive tumor progression by increasing ROS generation, thereby triggering oxidative DNA damage and pro-inflammatory signaling cascades 45. Collectively, our findings reveal a previously unrecognized mechanism whereby mtPD-L1 translocation rewires mitochondrial energy metabolism and translation, thereby promoting HBx-driven HCC progression. Targeting mtPD-L1 offers a potential strategy to disrupt tumor-cell energy supply and improve responsiveness to immune checkpoint blockade in HBx-expressing HCC.

Beyond its canonical immune checkpoint role, mtPD-L1 operates as a metabolic regulator that drives non-immune functions. Here, we show that mtPD-L1 activates the mTOR/PGC-1α axis to promote mitochondrial biogenesis, thereby increasing ATP and NAD^+^ availability to support tumor growth and stress adaptation. Importantly, this mtPD-L1-driven metabolic remodeling is also likely to reduce susceptibility to immune effector stress through two complementary mechanisms. First, by enhancing mitochondrial biogenesis, mitochondrial translation, oxidative phosphorylation capacity, and redox buffering, mtPD-L1 increases tumor-cell fitness under immune pressure, thereby improving tolerance to immune effector-associated cytotoxic stress. Second, heightened mitochondrial activity may indirectly constrain effector T-cell function by intensifying nutrient and oxygen competition within the tumor microenvironment and by promoting accumulation of immunosuppressive metabolites. Accordingly, αPD-L1 monotherapy is insufficient to suppress this mtPD-L1-dependent metabolic program, highlighting the need for combined therapeutic strategies. In this context, O-GlcNAcylation emerges as a molecular switch governing mtPD-L1 trafficking and subcellular localization. HBx-induced O-GlcNAcylation redirects PD-L1 trafficking from the Golgi to mitochondria, amplifying mitochondrial biogenesis and reinforcing immune-metabolic adaptation. Consistent with the clinical relevance of this pathway, IHC analysis of an ICB-treated HBV-HCC cohort (n = 10 pairs of tumor and adjacent tissue samples) showed significantly higher levels of PD-L1, global O-GlcNAcylation, PGC-1α, and mTOR in tumors than in paired adjacent tissues. Moreover, within tumor tissues, PD-L1 expression positively correlated with O-GlcNAcylation, PGC-1α, and mTOR, supporting a clinically relevant PD-L1-O-GlcNAc-mitochondrial biogenesis axis in HBV-HCC. Although direct assessment of mtPD-L1 and response-stratified analyses in ICB responders versus non-responders were limited by the current availability of predominantly FFPE specimens, these patient data provide translational support for our mechanistic findings. Pharmacologically, the OGT inhibitor OSMI-1 disrupted this pathogenic circuit, abolished the mitochondrial function of PD-L1, and sensitized HBx-expressing HCC tumors to αPD-L1 therapy. Importantly, OSMI-1 showed minimal intrinsic cytotoxicity and has demonstrated therapeutic potential across multiple disease models 46, 47. In HBx-Tg mice bearing HCC xenografts, combined treatment with OSMI-1 and αPD-L1 markedly reduced tumor volume, depleted mtPD-L1, and downregulated mitochondrial biogenesis proteins, thereby increasing immune effector-associated tumor suppression. Collectively, our study identifies a non-classical mechanism in which HBx-driven O-GlcNAcylation re-localizes PD-L1 to mitochondria, enabling HCC cells to tolerate immune effector-associated pressure while sustaining a mitochondrial metabolic program. Targeting this spatial reprogramming with OSMI-1 restores sensitivity to immunotherapy and provides a rationale for combining αPD-L1 with OGT inhibition in HBV-related HCC.

## Conclusion

In summary, we identify an HBx-driven, tumor-cell-intrinsic immunometabolic axis in which O-GlcNAcylation redirects PD-L1 to mitochondria, generating mtPD-L1 through enhanced Golgi-mitochondria communication and GOLPH3/Drp1-dependent coupling. This axis promotes mitochondrial translation, mitochondrial biogenesis, and metabolic rewiring via mTOR/PGC-1α, thereby sustaining HBx-expressing HCC cell fitness and conferring resistance to αPD-L1 therapy. OGT inhibition with OSMI-1 disrupts this pathway, depletes mtPD-L1, and restores sensitivity to PD-L1 blockade. Functionally, these findings suggest that targeting the O-GlcNAc-mtPD-L1 axis weakens the adaptive fitness of HBx-related tumor cells and increases their susceptibility to immune effector-associated pressure. These findings highlight the O-GlcNAc-mtPD-L1-GOLPH3/Drp1-mTOR/PGC-1α axis as a therapeutic vulnerability in HBV-related HCC and support further evaluation of OGT inhibition combined with PD-L1 blockade.

## Supplementary Material

Supplementary methods, figures and tables.

## Data Availability

The data supporting the findings of this study are available within the article and its supplementary materials. Additional data are available from the corresponding authors upon reasonable request.
