# Supplementary material for: Targeting Mitochondrial PD-L1 O-GlcNAcylation to Sensitize HBV-Related HCC to Immunotherapy: Modulating Golgi-mitochondrial Crosstalk and mTOR/PGC-1α-Driven Mitochondrial Biogenesis to Overcome Resistance
Source: Int J Biol Sci. 2026 Jul 22;22(13):6892–913. doi: 10.7150/ijbs.126948 (PMC13540441; doi:10.7150/ijbs.126948)
Supplement: Supplementary file 1 [file ijbsv22p6892s1.pdf]

Supplementary materials

Targeting Mitochondrial PD-L1 O-GlcNAcylation to Sensitize HBV-Related HCC to Immunotherapy: Modulating Golgi–mitochondrial Crosstalk and mTOR/PGC-1α-Driven Mitochondrial Biogenesis to Overcome Resistance

Ze-Bang Du [1†], Xin-Yu Ma [1†], Han-Yu Zhang [1†], Jia-Ming Lei [1], Xin-Xin He [1], Wen-Qi Xu [1], Wen-Dan Zhou [1], Xiao-Gang Xia [2], Ao-Bo Zhuang [2], Xiao-Xuan Chen [1], You-Liang Yao [1], Wen-Gang Li [2*], Yu-Chun Lin [1*], and Zhong-Ning Lin [1*]

1 State Key Laboratory of Vaccines for Infectious Diseases, Xiang An Biomedicine Laboratory, Xiang'an Hospital of Xiamen University, National Innovation Platform for Industry-Education Integration in Vaccine Research, School of Public Health, Xiamen University, Xiamen 361102, China

2 Department of Hepatobiliary Surgery, Cancer Research Center, Xiang'an Hospital of Xiamen University, School of Medicine, Xiamen University, Xiamen, Fujian 361102, China.

[†] These authors contributed equally to this work.

[*] Corresponding author: lwgang@xmu.edu.cn (W.-G. Li); linych@xmu.edu.cn (Y.-C. Lin); and linzhn@xmu.edu.cn (Z.-N. Lin)

Tel: +86 592 2880615; Fax: +86 592 2881578.

Supplementary materials and methods

Antibodies and reagents

Dulbecco's Modified Eagle Medium (DMEM), RPMI-1640, 0.25% trypsin-EDTA, and penicillin/streptomycin were purchased from Gibco (Grand Island, NY, USA). Fetal bovine serum (FBS) was obtained from ExCell Bio (Taicang, Jiangsu, China). Primary antibodies against β-actin, Na^+/K^+-ATPase, GOLPH3, Drp1, PD-L1, TOM20, mTOR, p-mTOR^{Ser2448}, and OGT were purchased from ABclonal (Wuhan, Hubei, China). Antibodies against granzyme B (GZMB) and CD8α were purchased from Abmart (Shanghai, China). Antibodies against HBx, COX IV, O-GlcNAc (CTD110.6), and PCNA were purchased from Santa Cruz Biotechnology (Dallas, TX, USA). Antibodies against PGC-1α, NRF1, and TFAM were purchased from Affinity Biosciences (Changzhou, Jiangsu, China). Horseradish peroxidase (HRP)-conjugated secondary antibodies were obtained from Cell Signaling Technology (Danvers, MA, USA). OSMI-1, PUGNAc, and tunicamycin (TM) were purchased from MedChemExpress (Monmouth Junction, NJ, USA); rapamycin (RAPA), MHY1485, and SR-18292 were purchased from Selleck Chemicals (Houston, TX, USA); chloramphenicol (CL) was purchased from Sigma-Aldrich (St. Louis, MO, USA).

Plasmid construction and gene editing

HepG2 cells were transfected with pcDNA3.1-*HBx* to generate HBx-expressing cells, whereas HepG2.2.15 cells were transfected with PB-20F3 transposon plasmids encoding an anti-HBx antibody (kindly provided by Prof. Quan Yuan, Xiamen University) to achieve HBx knockdown [1]. For mitochondrial PD-L1 (mtPD-L1) overexpression, MHCC-97H and HepG2.2.15 cells were infected with pCDH-MTS-*CD274* lentivirus as described [2]. CRISPR/Cas9-mediated PD-L1 knockdown in HepG2.2.15 cells was performed using a Cas9-*CD274* plasmid. PD-L1 O-GlcNAcylation-deficient mutants (S283A/T285A) and shDNM1L plasmids for Drp1 knockdown were purchased from MiaoLingPlasmid (Wuhan, Hubei, China). GOLPH3 was depleted using a Cas9-*GOLPH3* plasmid or reconstituted with either wild-type or R90L mutant plasmids (MiaoLingPlasmid) to assess dependence on PI(4)P binding. All plasmids and primer sequences are listed in Supplementary Table S4.

Cell culture and treatments

Human HCC cell lines (HuH-7, HepG2, BEL-7404, SMMC-7721, PLC/PRF/5, Hep3B, MHCC-97H, HepG2.2.15) were maintained in our laboratory and cultured in complete DMEM supplemented with 10% FBS and 1% penicillin/streptomycin at 37 °C in 5% CO_2, as previously described [3]. Human peripheral blood mononuclear cells (PBMCs) were isolated from healthy adult donors by Ficoll-Paque PLUS density-gradient centrifugation (GE Healthcare, Chicago, IL, USA) and cultured in high-glucose RPMI-1640 containing 10% FBS and 30 IU/mL recombinant human IL-2 (PeproTech, Rocky Hill, NJ, USA).

N-linked glycosylation was inhibited with tunicamycin (TM, 1 μg/mL, 24 h). O-GlcNAcylation was suppressed with OSMI-1 (10 μM, 24 h) or enhanced with PUGNAc (10 μM, 24 h). Mitochondrial translation was blocked using chloramphenicol (CL, 100 μg/mL, 24 h); mTOR signaling was inhibited with rapamycin (RAPA, 100 nM, 24 h) or activated with MHY1485 (5 μM, 24 h). To assess mtPD-L1-driven mitochondrial biogenesis, cells were treated with SR-18292 (10 μM, 24 h).

Functional immune assays were performed as described [4]. Tumor cells (2.5×10^4 per well) were co-cultured with pre-activated PBMCs (an effector-to-target ratio of 5:1) in the presence or absence of anti-PD-L1 antibody (αPD-L1, 10 μg/mL, clone 29E.2A3, BioLegend, San Diego, CA, USA) for 24 h. Cytotoxicity and cytokine release were quantified using an LDH Cytotoxicity Assay (Beyotime, Shanghai, China), a human IFN-γ ELISA (Yisheng Biotechnology, Hangzhou, Zhejiang, China), and a human TNF-α ELISA (Linko Biotechnology, Hangzhou, Zhejiang, China).

Histopathology and Immunohistochemistry (IHC)

Tumor tissues were fixed in 4% (w/v) paraformaldehyde at 4 ℃ overnight, paraffin-embedded, and sectioned. Hematoxylin and eosin (H&E) staining was performed using standard protocols. For IHC, antigen-retrieved sections were incubated with primary antibodies against Ki67, PCNA, PGC-1α, or O-GlcNAc, followed by HRP-conjugated polymer detection with commercial IHC kits (Maxim, Fuzhou, Fujian, China) and visualization with DAB. To ensure rigorous histopathological evaluation, all IHC images presented in this study were replaced with high-resolution original images exported directly from the acquisition software and standardized to publication-quality settings. Brightness and contrast were adjusted

uniformly across groups without altering the underlying signal. Representative fields were selected at appropriate magnification with clearly visible scale bars and legible labels. In addition, quantitative analyses of IHC signals, including signal intensity, positive area, or cell counts as appropriate, were incorporated to support the visual observations. Detailed antibody information is listed in Supplementary Table S2.

Transmission electron microscopy (TEM)

TEM was performed as previously described [5]. Cells were fixed with 0.1 M glutaraldehyde buffer, postfixed with 1% osmium tetroxide, dehydrated, embedded, and sectioned into ultrathin slices at the electron microscopy facility. The ultrastructure of mitochondria and the Golgi apparatus was examined with a Tecnai 20 transmission electron microscope (FEI, USA).

EdU proliferation assay

Cells (500 per well) were seeded onto glass coverslips in 24-well plates and incubated with EdU working solution for 2 h at 37 °C using the BeyoClick™ EdU Cell Proliferation Kit (Beyotime). After fixation in 4% paraformaldehyde (30 min, RT) and permeabilization, the Click reaction was performed (30 min, RT, protected from light). Nuclei were counterstained with Hoechst 33342, coverslips were mounted in glycerol, and images were acquired on a laser scanning confocal microscope (Zeiss LSM 780, Oberkochen, Germany).

Colony formation assay

For colony formation assays, 500 cells were seeded per well in 6-well plates and cultured for 7 days [6]. Colonies were then fixed with 4% paraformaldehyde (15 min, RT), stained with 0.1% crystal violet solution (15 min), rinsed, air-dried, and quantified using ImageJ (Bethesda, MD, USA).

Migration assay (scratch-wound)

Cells were grown to 90–100% confluence in 6-well plates. A linear scratch was created with a sterile 200-μL pipette tip, and detached cells were removed by washing twice with PBS. Cells were then cultured in low-serum medium (1% FBS) [7], and wound closure was imaged at 0, 12, and 24 h using an inverted microscope (TS-100, Nikon, Tokyo, Japan).

Apoptosis analysis by flow cytometry

Apoptosis was evaluated using an Annexin V-FITC/PI kit (Beyotime) according to the

manufacturer's protocol [5]. Briefly, cells were collected, washed with ice-cold PBS, resuspended in 195 μL Annexin V binding buffer, and incubated with 5 μL Annexin V-FITC and 10 μL propidium iodide (PI) for 20 min at room temperature (RT) in the dark. Samples were analyzed on a flow cytometer (Beckman Coulter, Indianapolis, IN, USA) and data were processed with FlowJo v10.8.

Cytotoxicity assays

LDH release and cell viability were measured with an LDH Cytotoxicity Assay Kit and a Calcein-AM/PI Kit (Beyotime), respectively. For the LDH assay, cells grown to 80–90% confluence in 96-well plates were incubated in low-serum medium (1% FBS), and maximum-release wells were treated with 10% lysis reagent. After centrifugation (400 × g, 5 min), 120 μL of supernatant was mixed with 60 μL of LDH detection reagent (30 min, protected from light), followed by absorbance measurement at 490 nm. For the Calcein-AM/PI assay, cells were incubated with staining solution for 20 min at 37 °C in the dark, and fluorescence was measured using a CLARIOstar microplate reader (BMG Labtech, Ortenberg, Germany).

ATP and NAD^+/NADH assays

ATP levels and NAD^+/NADH ratios were measured using colorimetric assay kits (Beyotime) according to the manufacturer's instructions. Absorbance was measured with a CLARIOstar microplate reader (BMG Labtech).

Immunofluorescence (IF) assay

Cells grown on glass coverslips were fixed with 4% paraformaldehyde, permeabilized with 0.5% Triton X-100, and blocked with 5% BSA in PBS. Samples were then incubated with primary antibodies overnight at 4 °C, followed by fluorophore-conjugated secondary antibodies (1 h, RT, protected from light) [5]. Nuclei were counterstained with DAPI, and coverslips were mounted in glycerol. Images were acquired using a laser scanning confocal microscope (Zeiss LSM 780). To support representative immunofluorescence observations with quantitative evidence, fluorescence signals were quantified as mean fluorescence intensity and/or integrated density per cell or per field, as appropriate. Colocalization was further assessed by line-scan intensity profiling, and PLA signals were quantified as puncta per cell. All image analyses were performed in a blinded manner using multiple randomly selected fields per sample, with identical acquisition settings and uniform analysis thresholds across groups. Antibody details

are listed in Supplementary Table S3.

Mitochondrial, membrane and cytosolic fractionation

Mitochondria and cytosol were isolated with the Cell Mitochondria Isolation Kit (Beyotime) according to the manufacturer's instructions. HBx-expressing HCC cells were resuspended in ice-cold mitochondrial lysis buffer for 10 min and homogenized with 40 strokes using a pre-chilled glass homogenizer. The homogenate was centrifuged at 600 × g for 10 min at 4 °C to remove nuclei and debris; the supernatant was further centrifuged at 12,000 × g for 10 min at 4 °C to pellet mitochondria. The resulting supernatant was collected as the cytosolic fraction and the mitochondrial pellet was resuspended for WB or mitochondrial immunoprecipitation (Mito-IP). COX IV was used as a mitochondrial marker to verify mitochondrial enrichment and, where applicable, as a loading control. Fraction purity was further assessed by immunoblotting with compartment-specific markers, including Na^+/K^+-ATPase (plasma membrane), β-actin (cytosol), histone H3 (nucleus), and ER/Golgi markers when applicable. These controls confirmed robust COX-IV enrichment with minimal to undetectable non-mitochondrial signals in the mitochondrial fraction, excluding plasma membrane contamination as the source of the mtPD-L1 signal.

For membrane fractionation, the post-nuclear supernatant was centrifuged at 14,000 × g for 30 min at 4 °C to pellet membrane fragments. The supernatant was collected as the cytosolic protein fraction, whereas the membrane pellet was briefly re-centrifuged, residual supernatant was carefully removed, and the pellet was resuspended in membrane protein extraction buffer B (200–300 μL) by vigorous vortexing, followed by incubation on ice for 5–10 min; this vortex/ice incubation cycle was repeated 1–2 times to ensure efficient membrane protein extraction. After centrifugation at 14,000 × g for 5 min at 4 °C, the supernatant was collected as the membrane protein fraction.

Quantitative real-time PCR (qRT-PCR)

Total RNA was extracted with TRIzol reagent and reverse-transcribed using the PrimeScript™ RT Reagent Kit (Takara, Tokyo, Japan), as previously described [8]. Quantitative real-time PCR was performed using iTaq™ Universal SYBR® Green Supermix on a qTOWER 3G system (Jena, Germany). mRNA levels were normalized to *ACTB* and calculated using the $2^{-\Delta\Delta Ct}$ method. All primer sequences are listed in Supplementary Table S4.

mtDNA copy number

Genomic DNA was isolated using the Universal Genomic DNA Purification Mini Spin Kit (Beyotime) according to the manufacturer's instructions. mtDNA content was quantified by qPCR targeting the mitochondrial D-loop region, using 18S rDNA as the nuclear reference. Primer sequences were as follows: D-loop forward: 5'-CAAACCTACGCCAAAATCCA-3', and D-loop reverse: 5'-GAAATGAATGAGCCTACAGA-3'; 18S rDNA forward: 5'-ACGGACCAGAGCGAAAGCA-3', and 18S rDNA reverse: 5'-GACATCTAAGGGCATCACAGAC-3'.

Western blotting (WB) and immunoprecipitation (IP)

Cells were lysed in SDS lysis buffer, and proteins were separated by SDS-PAGE and transferred onto PVDF membranes (Millipore, Burlington, MA, USA) as previously described. Membranes were blocked with 5% (w/v) non-fat milk in TBST, incubated with primary antibodies (overnight, 4 °C), and then with HRP-conjugated secondary antibodies (1 h, RT). Signals were detected using an enhanced chemiluminescence (ECL) kit (ABclonal) and captured with an Azure Biosystems imager (Dublin, CA, USA). For the IP assay, lysates were incubated with the indicated antibody and Protein A/G magnetic beads (MedChemExpress, Rahway, NJ, USA) as described [5]. Antibody details are listed in Supplementary Table S5.

Supplementary References

1. Du ZB, Wu XM, Han T, Cai YX, Qian B, Shen YS, et al. MALAT1 promotes malignancy of HBV-related hepatocellular carcinoma by regulating IGF2BP3-mediated nuclear-cytoplasmic shuttling. Int J Biol Sci. 2025; 21: 4942-60.
2. Li T, Huang HY, Qian B, Wang WH, Yuan Q, Zhang HY, et al. Interventing mitochondrial PD-L1 suppressed IFN-gamma-induced cancer stemness in hepatocellular carcinoma by sensitizing sorafenib-induced ferroptosis. Free Radic Biol Med. 2024; 212: 360-74.
3. Che L, Huang J, Lin JX, Xu CY, Wu XM, Du ZB, et al. Aflatoxin B1 exposure triggers hepatic lipotoxicity via p53 and perilipin 2 interaction-mediated mitochondria-lipid droplet contacts: An in vitro and in vivo assessment. J Hazard Mater. 2023; 445: 130584.
4. Liao P, Wang W, Wang W, Kryczek I, Li X, Bian Y, et al. CD8(+) T cells and fatty acids orchestrate tumor ferroptosis and immunity via ACSL4. Cancer Cell. 2022; 40: 365-378.e6.
5. Che L, Wu JS, Xu CY, Cai YX, Lin JX, Du ZB, et al. Protein phosphatase 2A-B56gamma-Drp1-Rab7 signaling axis regulates mitochondria-lysosome crosstalk to sensitize the anti-cancer therapy of hepatocellular carcinoma. Biochem Pharmacol. 2022; 202: 115132.
6. Wang Z, He K, Liu M, Lv W, Cheng B, Zhang G, et al. Enhanced mitochondrial biogenesis facilitates the

development of cutaneous squamous cell carcinoma. Cancer Lett. 2025; 618: 217623.
7. Zhang D, Man D, Lu J, Jiang Y, Ding B, Su R, et al. Mitochondrial TSPO Promotes Hepatocellular Carcinoma Progression through Ferroptosis Inhibition and Immune Evasion. Adv Sci (Weinh). 2023; 10: e2206669.
8. Chen YY, Wang WH, Che L, Lan Y, Zhang LY, Zhan DL, et al. BNIP3L-Dependent Mitophagy Promotes HBx-Induced Cancer Stemness of Hepatocellular Carcinoma Cells via Glycolysis Metabolism Reprogramming. Cancers (Basel). 2020; 12: 655.

Supplementary figures and legends

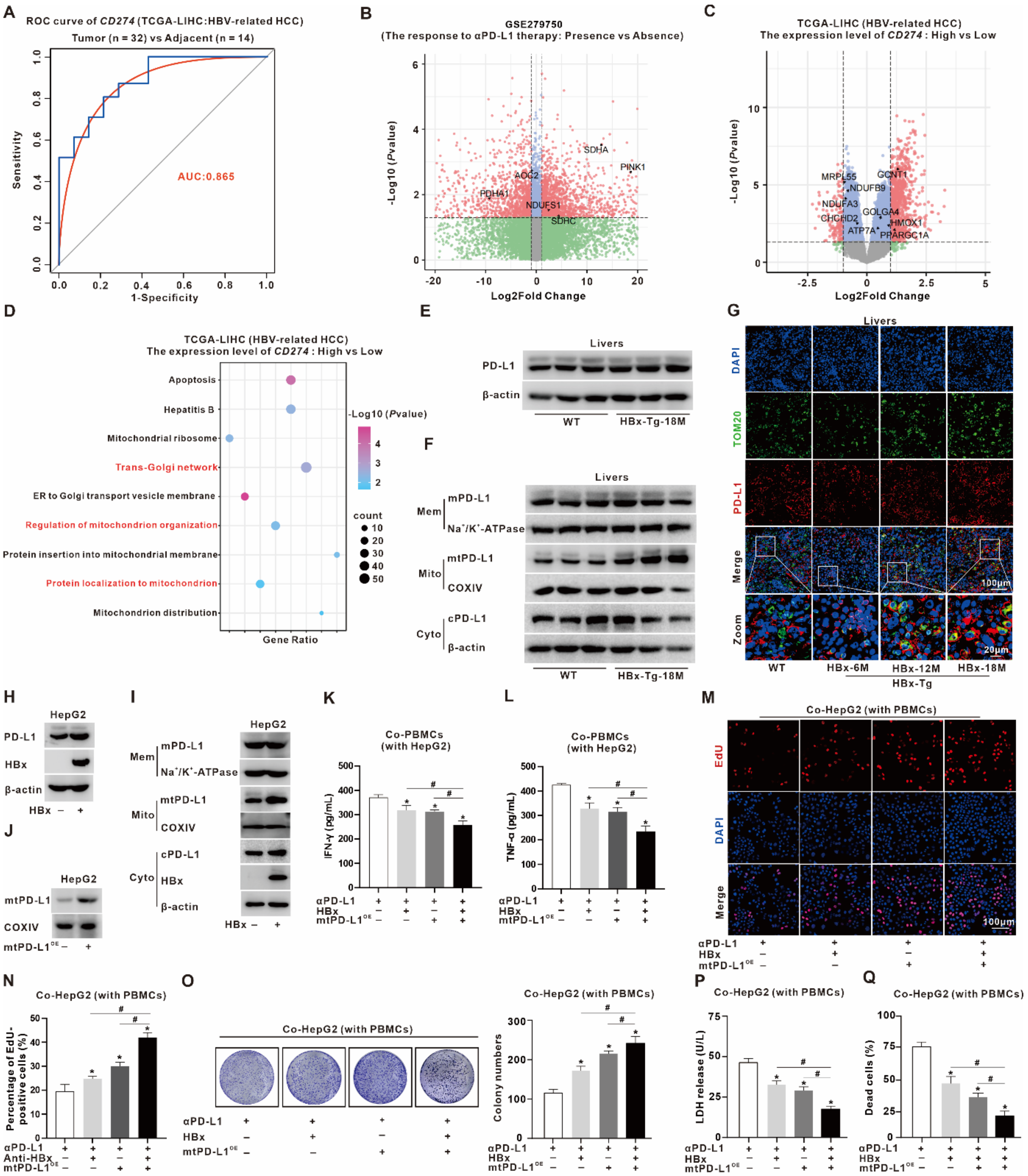

Fig. S1. HBx-driven mtPD-L1 orchestrates resistance to αPD-L1-associated immune

pressure in HCC. (A) ROC curve assessing *CD274* as a diagnostic marker to distinguish HBV-related HCC tumors from adjacent tissues in the TCGA-LIHC cohort. (B) The volcano plot of differentially expressed genes (DEGs) in HCC patients treated with αPD-L1 therapy based on GSE279750. (C) The volcano plot of DEGs in HBV-related HCC patients from the TCGA-LIHC cohort. (D) GO enrichment analysis of DEGs between *CD274*-high and *CD274*-low HBV-related HCC patients in the TCGA-LIHC cohort. (E–G) Liver samples from C57BL/6 wild-type (WT) and HBx-Tg mice aged 6, 12, or 18 months. n = 3 per group. (E) Total PD-L1 levels in liver lysates. (F) PD-L1 levels in subcellular fractions (membrane, mitochondria, and cytosol) from liver tissues. (G) Representative IF images of TOM20 (a mitochondrial marker) and PD-L1 staining in liver sections. Scale bar: 10 μm. (H–I) HBx-expressing HepG2 cells were generated by transfection with pcDNA3.1-*HBx* for 24 h, with empty vector serving as a negative control. (H) Total PD-L1 levels were detected by WB. (I) PD-L1 levels in subcellular fractions (membrane, mitochondria, and cytosol) were detected by WB. (J–Q) HepG2 cells overexpressing mitochondria-targeted PD-L1 (mtPD-L1^{OE}), generated by fusing PD-L1 to a mitochondrial targeting sequence (MTS), and transfected with pcDNA3.1-*HBx*, were co-cultured with activated PBMCs and treated with or without αPD-L1 (10 μg/mL, 24 h). (J) Validation of mtPD-L1^{OE} HepG2 cells. (K–L) PBMC-derived IFN-γ (K) and TNF-α (L) levels were measured by ELISA. (M) Representative images of EdU incorporation assays. Scale bar: 100 μm. (N) Quantification of EdU-positive cells is shown in the bar graph. (O) Representative images (left) and quantification (right) of colony formation. (P–Q) LDH release (P) and dead-cell percentage (Q). Data are mean ± SD, n = 3. *, $P < 0.05$ versus control; #, $P < 0.05$ versus the indicated group.

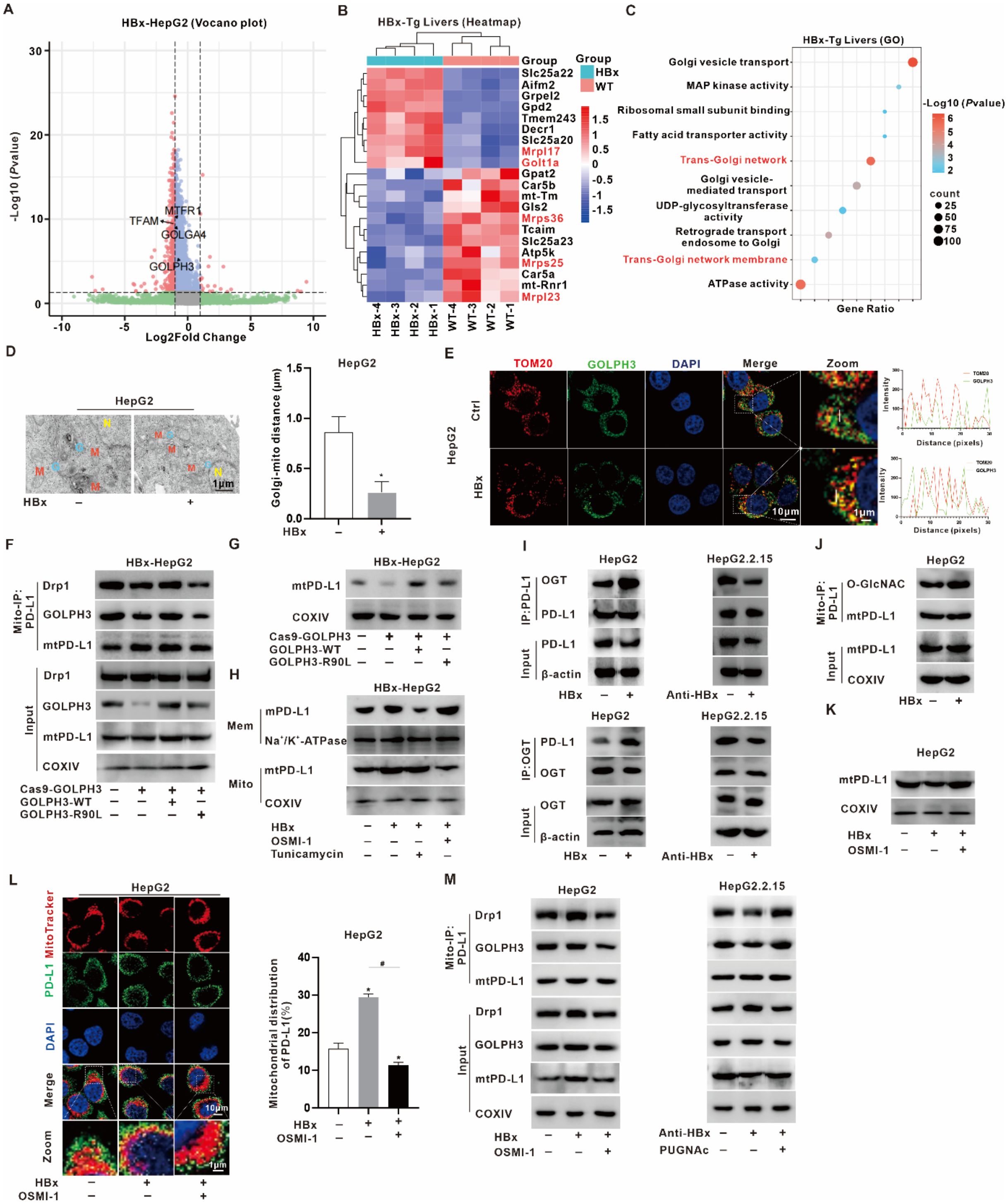

Fig. S2. O-GlcNAcylation of PD-L1 drives its mitochondrial translocation via remodeling Golgi–mitochondria communication in HBx-expressing HCC cells. (A) The volcano plot of DEGs from RNA-sequencing transcriptomic analysis of HBx-expressing HepG2 cells (n = 3). (B–C) Livers from HBx-transgenic (HBx-Tg) mice were used for RNA sequencing; wild-type (WT) C57BL/6 mice served as controls (n = 4 per group). (B) Heatmap showing DEGs associated with mitochondria or the Golgi apparatus. (C) GO enrichment analysis of DEGs. (D)

Representative TEM images showing the proximity between the Golgi apparatus and mitochondria in HBx-expressing HepG2 cells (upper); quantification of the shortest Golgi–mitochondria distance (μm) is shown in the bar graph (lower). G, Golgi apparatus; M, mitochondrion; N, nucleus. Scale bar: 1 μm. (E) Representative IF images showing TOM20 (mitochondrial marker, green) and GOLPH3 (Golgi marker, red) co-staining in HBx-expressing HepG2 cells. Nuclei were counterstained with DAPI (blue) (left); line-scan intensity profiles are shown to illustrate signal co-distribution (right). Scale bar: 10 μm (main) and 1 μm (Zoom). (F–G) CRISPR/Cas9-mediated GOLPH3 knockdown (Cas9-GOLPH3) was performed in HBx-expressing HepG2 cells, followed by transfection with wild-type (GOLPH3-WT) or R90L mutant (GOLPH3-R90L) plasmids for 24 h. (F) Mito-IP assessing the interaction between mtPD-L1 and the GOLPH3/Drp1 complex. (G) mtPD-L1 levels in mitochondrial fractions were detected by WB. (H) HBx-expressing HepG2 cells were treated with OSMI-1 (10 μM) or tunicamycin (TM, 1 μg/mL) for 24 h. Membrane and mitochondrial PD-L1 levels were then detected by WB. (I) Co-IP assessing the interaction between PD-L1 and OGT in HBx-expressing HepG2 cells (left) and anti-HBx-treated HepG2.2.15 cells (right). (J) Mito-IP assessing the O-GlcNAcylation status of mtPD-L1. (K–L) HBx-expressing HepG2 cells were treated for 24 h with OSMI-1 (10 μM). (K) mtPD-L1 levels in mitochondrial fractions. (L) Representative IF images showing mtPD-L1 (green) localization relative to mitochondria (MitoTracker, red). Nuclei were counterstained with DAPI (blue) (left); line-scan intensity profiles are shown to illustrate signal co-distribution (right). Scale bar: 10 μm (main) and 1 μm (Zoom). (M) HBx-expressing HepG2 cells (left) and anti-HBx-treated HepG2.2.15 cells (right) were treated for 24 h with OSMI-1 (10 μM) or PUGNAc (10 μM). Mito-IP assessing the interaction between mtPD-L1 and the GOLPH3/Drp1 complex. Data are mean ± SD, n = 3. *, $P < 0.05$ versus control; #, $P < 0.05$ versus the indicated group.

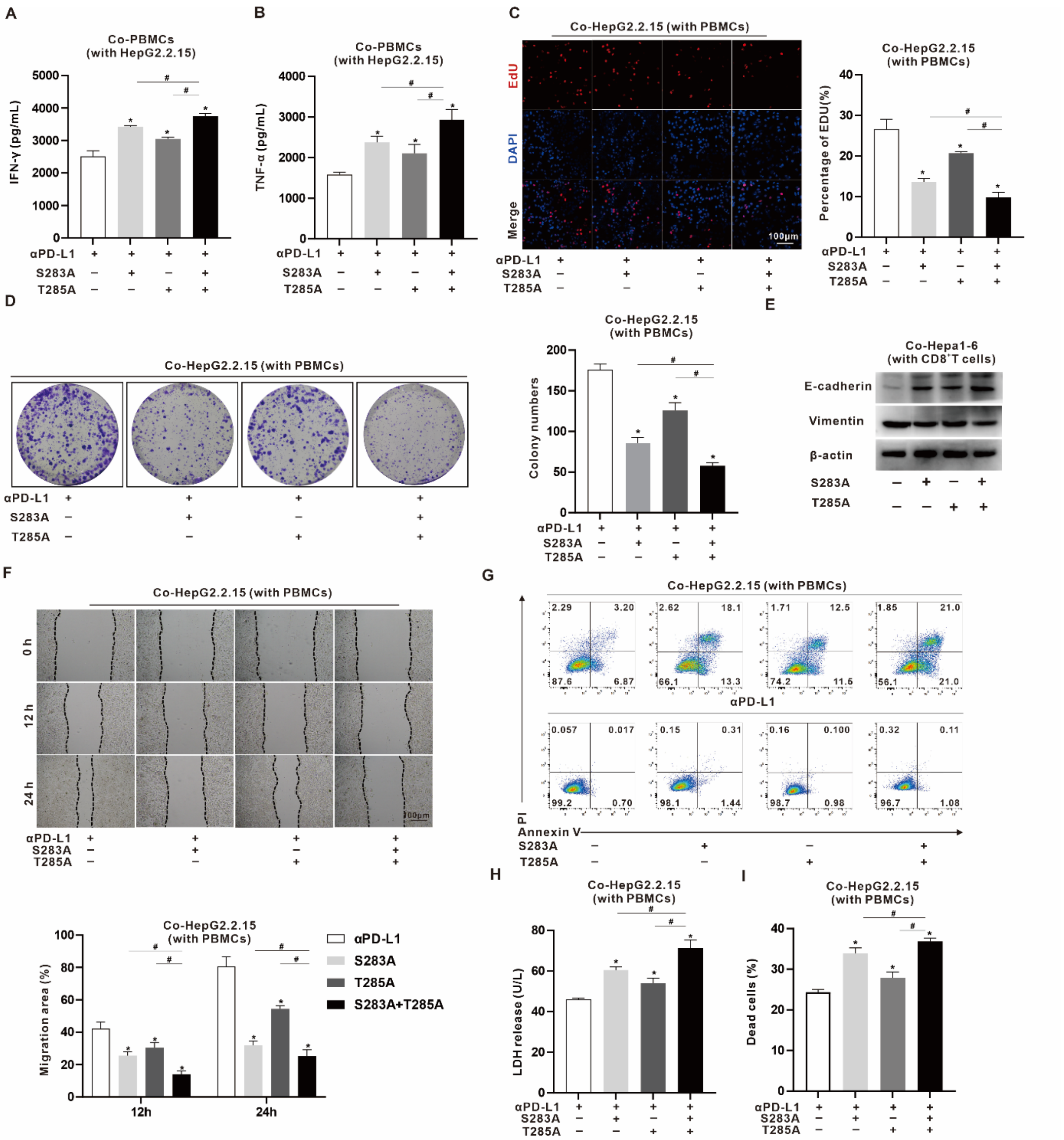

Fig. S3. OGT-mediated O-GlcNAcylation of PD-L1 facilitates its mitochondrial localization and promotes immune pressure-resistant phenotypes in HepG2.2.15 cells. (A–I) HepG2.2.15 cells expressing PD-L1 (WT) or the indicated O-GlcNAcylation-site mutants (S283A, T285A, or S283A/T285A) were co-cultured with activated PBMCs and treated with anti-PD-L1 antibody (αPD-L1, 10 μg/mL, 24 h) *in vitro*. (A–B) PBMC-derived IFN-γ (A) and TNF-α (B) levels were measured by ELISA as cytokine readouts in the PBMC–tumor co-culture system. (C) Representative IF images of EdU incorporation assays for cell proliferation (left); the percentage of EdU-positive cells is presented in the bar graph (right); nuclei were

counterstained with DAPI. Scale bar: 100 μm. (D) Representative images of colony formation assays (left); quantification of colony numbers is presented in the bar graph (right). (E) Levels of cell migration-related proteins E-cadherin and Vimentin. (F) Representative images of scratch-wound assays evaluating cell migration at 0, 12, and 24 h (upper); quantification of migration rate is presented in the bar graph (lower). (G) Representative flow cytometry plots and quantification of apoptosis by Annexin V/PI staining. (H–I) LDH release (H) and percentage of dead cells (I) were measured. Data are mean ± SD, n = 3. *, $P < 0.05$ versus control; #, $P < 0.05$ versus the indicated group.

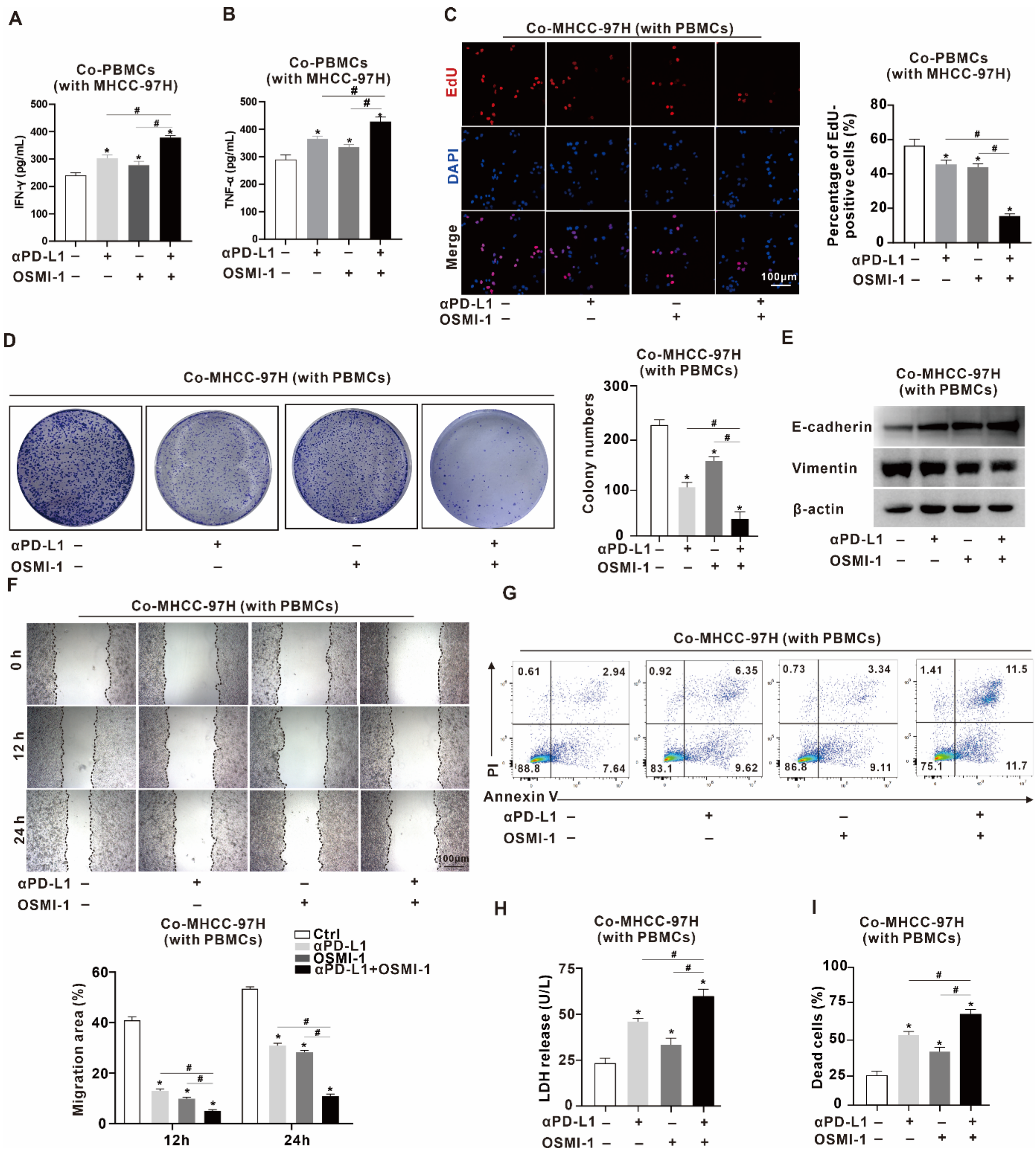

Fig. S4. OSMI-1 selectively targets O-GlcNAcylation and potentiates αPD-L1-associated tumor-cell suppression *in vitro*. Activated PBMCs and HBx-expressing MHCC-97H cells were co-cultured and treated with αPD-L1 (10 μg/mL), OSMI-1 (10 μM), or their combination for 24 h *in vitro*. (A–B) PBMC-derived IFN-γ (A) and TNF-α (B) levels were measured by ELISA. (C–I) Tumor phenotypes were assessed in the co-cultured MHCC-97H cells. (C) Representative IF images of EdU incorporation assays for cell proliferation (left); the percentage of EdU-positive cells is presented in the bar graph (right); nuclei were counterstained with DAPI. Scale bar: 100 μm. (D) Representative images of colony formation assays (left); quantification of colony numbers is presented in the bar graph (right). (E) Levels of cell migration-related proteins E-cadherin and Vimentin. (F) Representative images of scratch-wound assays evaluating cell migration at 0, 12, and 24 h (upper); quantification of migration rate is presented in the bar graph (lower). (G) Representative flow cytometry plots and quantification of apoptosis by Annexin V/PI staining. (H–I) LDH release (H) and the percentage of dead cells (I) were measured. Data are mean ± SD, n = 3. *, $P < 0.05$ versus control; #, $P < 0.05$ versus the indicated group.

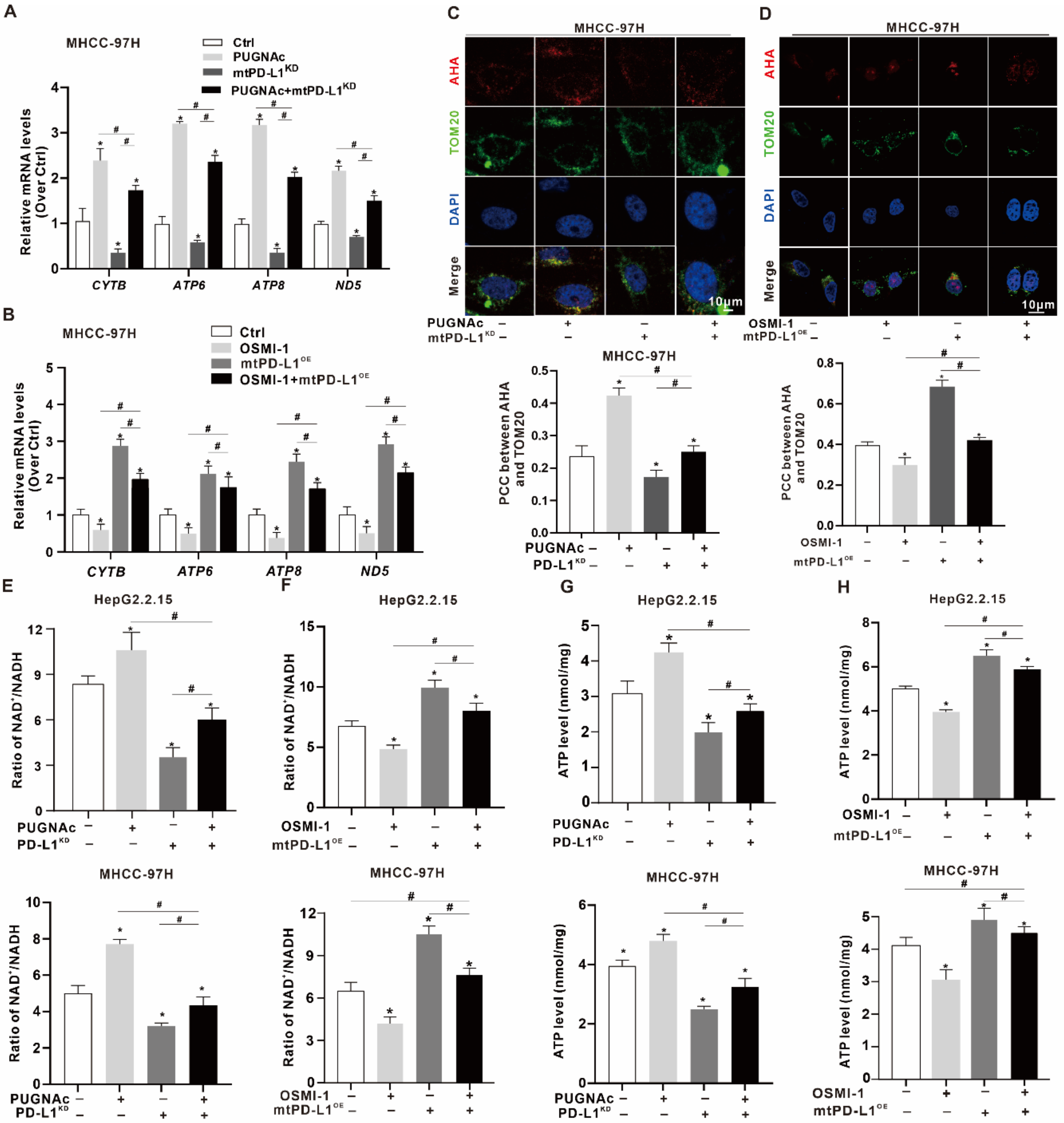

Fig. S5. O-GlcNAcylation-driven mtPD-L1 promotes mitochondrial translation and rewires energy metabolism in HBx-expressing HCC cells. PD-L1^{KD} MHCC-97H cells were treated with PUGNAc (10 μM), and mtPD-L1^{OE} MHCC-97H cells were treated with OSMI-1 (10 μM) or PUGNAc (10 μM). (A–B) Relative mRNA levels of mitochondrial-encoded genes were quantified by qRT-PCR after 12 h of treatment. (C–D) Representative IF images of AHA-labeled nascent mitochondrial proteins (fluorescence intensity) after 24 h of treatment (upper); quantification of AHA–TOM20 colocalization using Pearson's correlation coefficient (PCC) is presented in the bar graph (lower); nuclei were counterstained with DAPI. Scale bar: 10 μm. (E-H) NAD$^+$/NADH ratio (E–F) and intracellular ATP levels (G–H) were measured using the

NAD^+/NADH assay kit and ATP assay kit, respectively. Data are mean ± SD, n = 3. *, $P < 0.05$ versus control; #, $P < 0.05$ versus the indicated group.

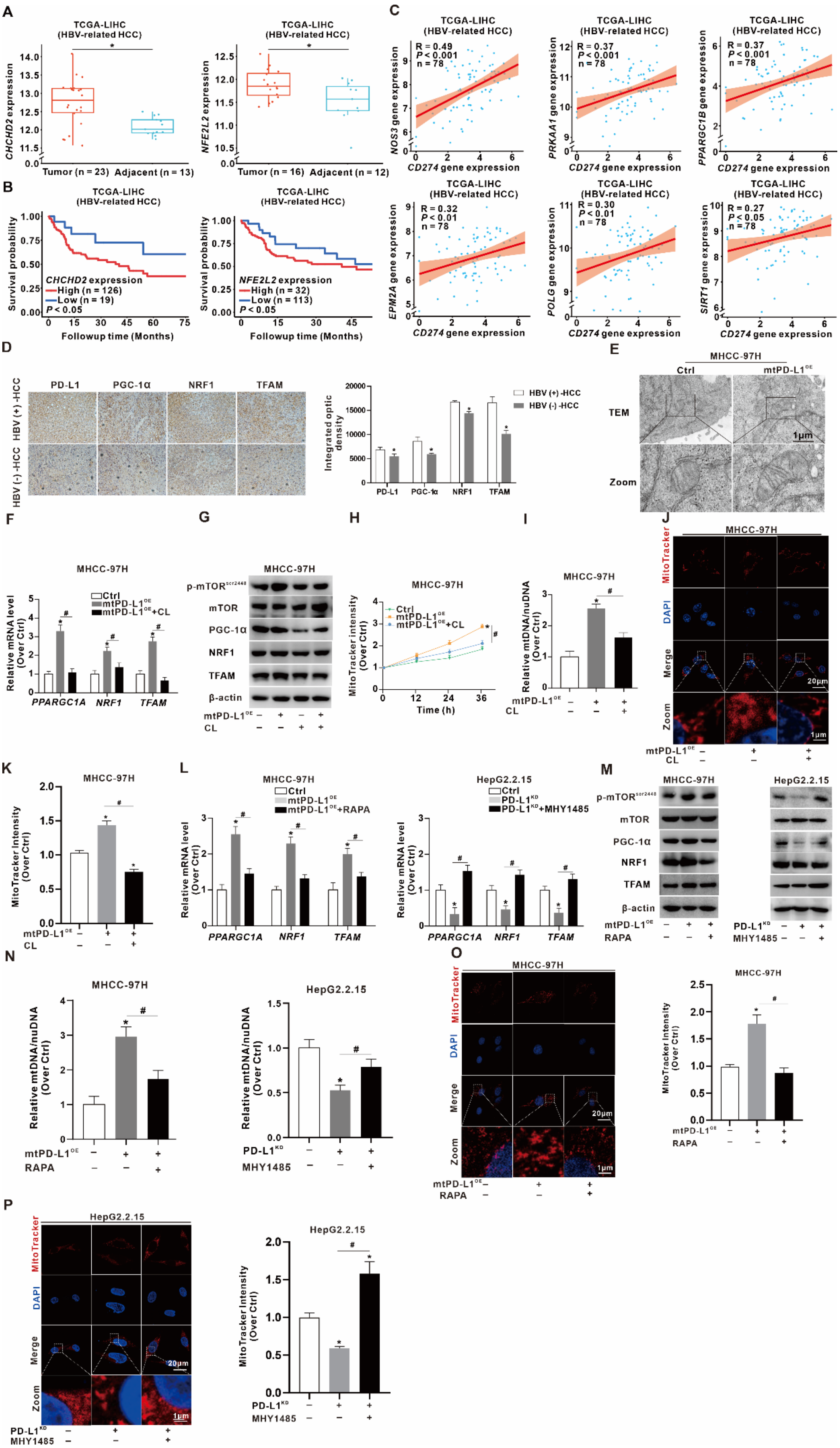
A
TCGA-LIHC (HBV-related HCC)
CHCHD2 expression
Tumor (n = 23) Adjacent (n = 13)
NFE2L2 expression
Tumor (n = 16) Adjacent (n = 12)
B
Survival probability
CHCHD2 expression
High (n = 126)
Low (n = 19)
P < 0.05
NFE2L2 expression
High (n = 32)
Low (n = 113)
Followup time (Months)
C
R = 0.49 P < 0.001 n = 78
NOS3 gene expression
R = 0.37 P < 0.001 n = 78
PRKAA1 gene expression
R = 0.37 P < 0.001 n = 78
PPARGC1B gene expression
R = 0.32 P < 0.01 n = 78
EPM2A gene expression
R = 0.30 P < 0.01 n = 78
POLG gene expression
R = 0.27 P < 0.05 n = 78
SIRT1 gene expression
CD274 gene expression
D
PD-L1 PGC-1α NRF1 TFAM
HBV (+) -HCC
HBV (-) -HCC
Integrated optic density
E
MHCC-97H
Ctrl mtPD-L1OE
TEM
Zoom
1μm
F
MHCC-97H
Relative mRNA level (Over Ctrl)
Ctrl
mtPD-L1OE
mtPD-L1OE+CL
PPARGC1A NRF1 TFAM
G
MHCC-97H
p-mTORser2448
mTOR
PGC-1α
NRF1
TFAM
β-actin
mtPD-L1OE
CL
H
MHCC-97H
MitoTracker intensity (Over Ctrl)
Time (h)
I
MHCC-97H
Relative mtDNA/nuDNA (Over Ctrl)
J
MHCC-97H
MitoTracker
DAPI
Merge
Zoom
20μm
1μm
K
MHCC-97H
MitoTracker Intensity (Over Ctrl)
L
MHCC-97H
mtPD-L1OE+RAPA
HepG2.2.15
PD-L1KD
PD-L1KD+MHY1485
M
RAPA
MHY1485
N
O
P
HepG2.2.15

Fig. S6. mtPD-L1 drives mitochondrial biogenesis via the mTOR/PGC-1α signaling axis in HBx-expressing HCC cells. (A) Box plots display the expression of mitochondrial biogenesis-related genes *CHCHD2* (left) and *NFE2L2* (right) in HBV-related HCC tumors and adjacent tissues from the TCGA-LIHC cohort. (B) Kaplan–Meier overall survival (OS) curves stratify HBV-related TCGA-LIHC patients by high versus low expression of *CHCHD2* (left) and *NFE2L2* (right). (C) Correlation analysis between *CD274* and mitochondrial biogenesis-related gene expression in tumor tissues from patients with HBV-related HCC (n = 78) in the TCGA-LIHC cohort. (D) Representative immunohistochemistry (IHC) images showing PD-L1 and mitochondrial biogenesis-related PGC-1α, NRF1, and TFAM protein levels in HBV(+) HCC and HBV(-) counterpart samples (n = 6) (left); quantification of PD-L1, PGC-1α, NRF1, and TFAM expression levels was performed using ImageJ software on IHC images (right). (E) Representative TEM images of mtPD-L1^{OE} MHCC-97H cells showing mitochondrial volume and morphology. Scale bar: 1 μm. (F–K) mtPD-L1^{OE} MHCC-97H cells were treated with or without chloramphenicol (CL, 100 μg/mL), a selective inhibitor of mitochondrial translation. (F) mRNA levels of mitochondrial biogenesis-related genes in cells treated for 12 h were quantified by qRT-PCR. (G) Levels of p-mTOR^{Ser2448}, total mTOR, and mitochondrial biogenesis-related proteins in cells treated for 24 h were detected by WB. (H) Quantification of mitochondrial biogenesis by flow cytometry analysis of MitoTracker fluorescence intensity in cells treated for 12, 24, and 36 h. (I) Quantification of mtDNA copy numbers in cells treated for 24 h, with nDNA copy numbers used as an internal reference. (J) Representative IF images of MitoTracker fluorescence intensity in cells treated for 24 h; nuclei were counterstained with DAPI. Scale bar: 20 μm (main) and 1 μm (Zoom). (K) Quantification of MitoTracker fluorescence intensity. (L–P) mtPD-L1^{OE} MHCC-97H cells were treated with rapamycin (RAPA, 100 nM) (left), or PD-L1^{KD} HepG2.2.15 cells were treated with MHY1485 (5 μM) (right). (L) mRNA levels of mitochondrial biogenesis-related genes in cells treated for 12 h were quantified by qRT-PCR. (M) Levels of p-mTOR^{Ser2448}, total mTOR, and mitochondrial biogenesis-related protein in cells treated for 24 h were detected by WB. (N) Quantification of mtDNA copy numbers in cells treated for 24 h, with nDNA copy numbers used as an internal reference. (O–P) Representative IF images of MitoTracker fluorescence intensity in cells treated for 24 h; nuclei were counterstained with DAPI (left); quantification of MitoTracker

fluorescence intensity (right). Scale bar: 20 μm (main) and 1 μm (Zoom). Data are mean ± SD, n = 3. *, $P < 0.05$ versus control; #, $P < 0.05$ versus the indicated group.

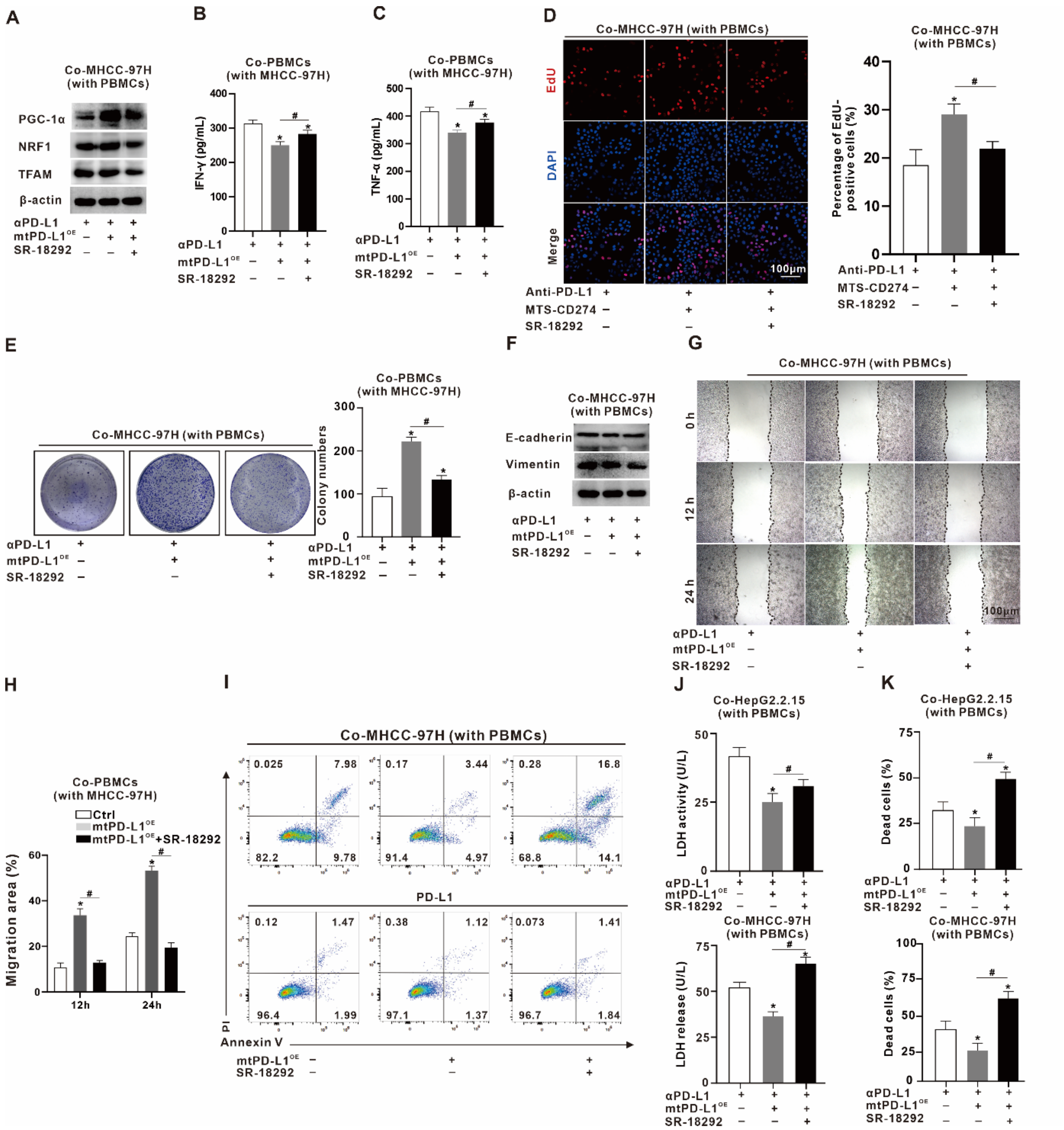

Fig. S7. mTOR/PGC-1α-dependent mitochondrial biogenesis governs the anti-tumor efficacy of αPD-L1 in HBx-expressing HCC cells. (A–K) mtPD-L1^{OE} MHCC-97H cells were co-cultured with activated PBMCs and treated with or without αPD-L1 (10 μg/mL) and/or SR-18292 (10 μM) for 24 h. (A) Levels of PGC-1α, NRF1, and TFAM proteins in MHCC-97H cells were detected by WB. (B–C) PBMC-derived IFN-γ (B) and TNF-α (C) levels were measured by ELISA. (D–J) Survival metrics of co-cultured mtPD-L1^{OE} MHCC-97H cells. (D)

Representative IF images from an EdU incorporation assay for cell proliferation (left); the percentage of EdU-positive cells is presented in the bar graph (right); nuclei were counterstained with DAPI. Scale bar: 100 µm. (E) Representative images of colony formation assays (left); quantification of colony numbers is presented in the bar graph (right). (F) Levels of cell migration-related proteins E-cadherin and Vimentin. (G) Representative images of scratch-wound assays evaluating cell migration at 0, 12, and 24 h. (H) Quantification of migration rate is presented in the bar graph. (I) Representative flow cytometry plots and quantification of apoptosis by Annexin V/PI staining. (J–K) LDH release (J) and the percentage of dead cells (K) were measured. Data are mean ± SD, n = 3. *, $P < 0.05$ versus control; #, $P < 0.05$ versus the indicated group.

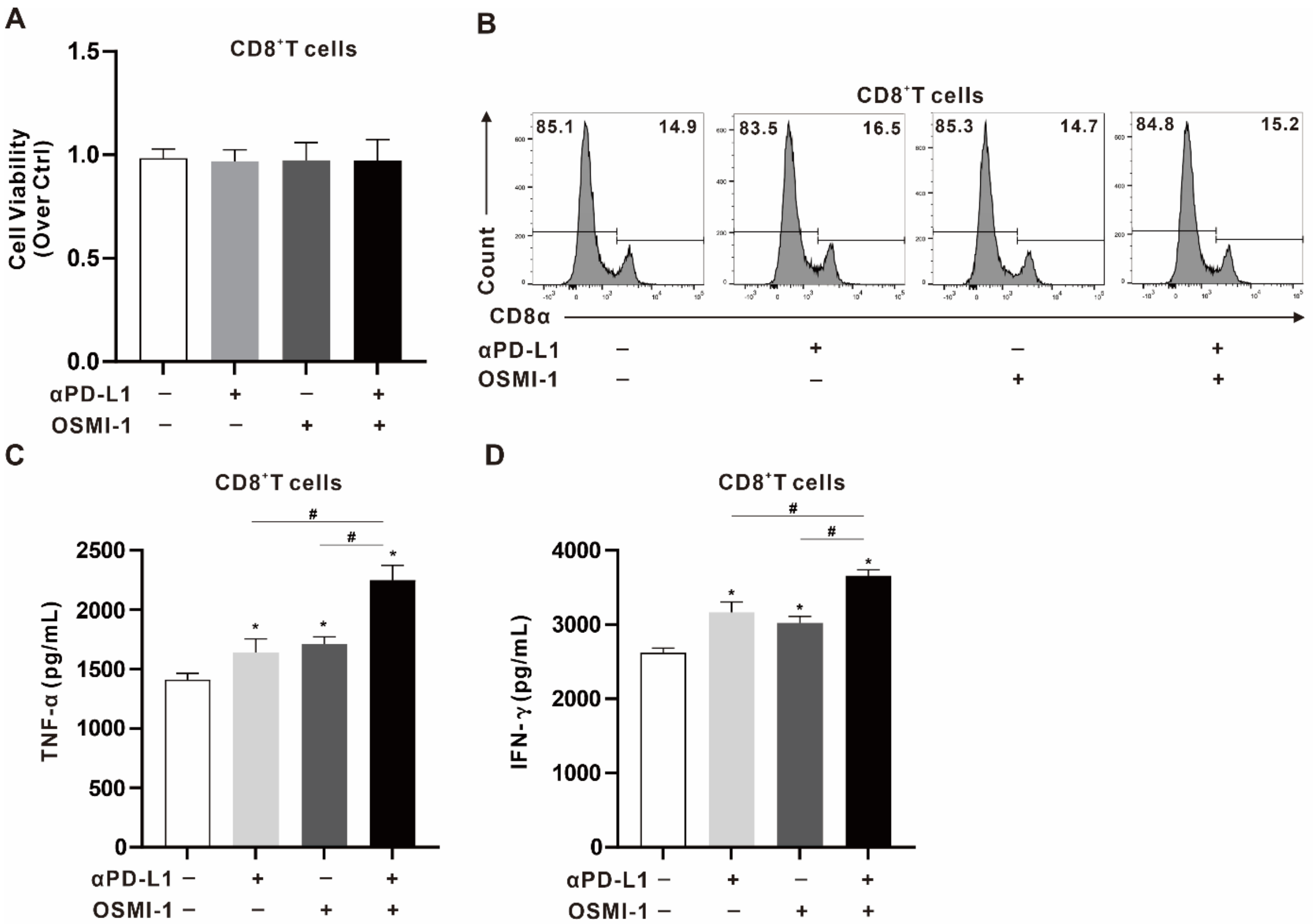

Fig. S8. OGT inhibition augments PD-L1 blockade-associated cytokine production by anti-CD3/CD28-preactivated CD8$^+$ T-cells without affecting cell viability or CD8α expression. (A) Viability of anti-CD3/CD28-preactivated CD8$^+$ T-cells following treatment with αPD-L1, OSMI-1, or their combination, presented as fold-change relative to the control group. (B) Representative flow-cytometric histograms of CD8α staining in CD8$^+$ T-cells under

the indicated treatments, showing comparable CD8α-positive populations across groups. (C–D) Levels of TNF-α (C) and IFN-γ (D) in culture supernatants of anti-CD3/CD28-preactivated CD8^{+} T-cells under the indicated treatments were measured by ELISA. Data are mean ± SD, n = 3. *, $P < 0.05$ versus control; #, $P < 0.05$ versus the indicated group.

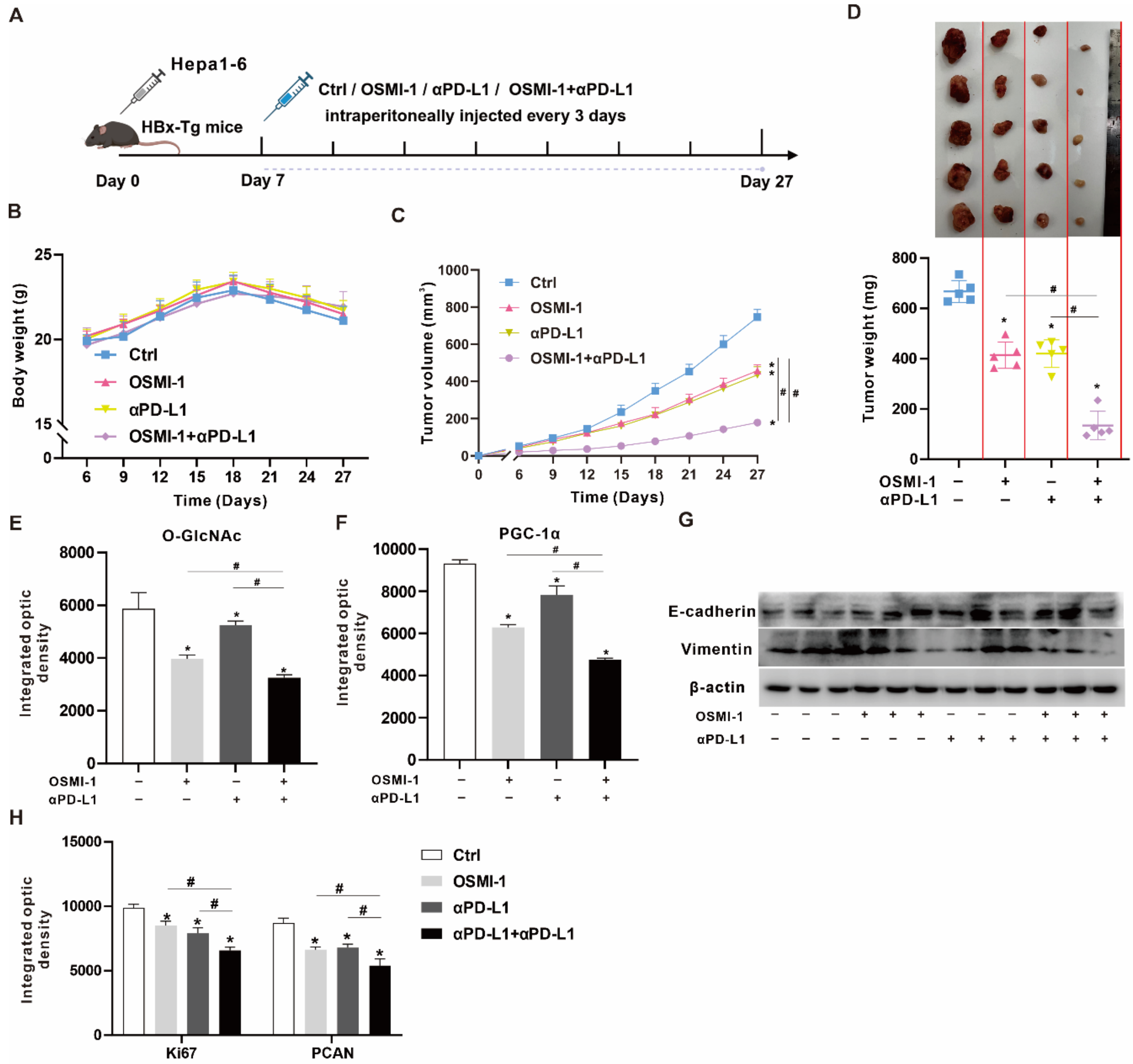

Fig. S9. OSMI-1 synergizes with αPD-L1 to overcome mtPD-L1-mediated resistance in HBV-driven HCC *in vivo*. (A) Schematic timeline of subcutaneous Hepa1-6 syngeneic tumor establishment in HBx-Tg mice and randomized treatment with vehicle (Ctrl), OSMI-1 (10 mg/kg, i.p.), αPD-L1 (5 mg/kg, i.p.), or the combination therapy administered by intraperitoneal injection every 3 days for 27 days. n = 5 per group. (B) Body weight curves of HBx-Tg mice monitored throughout the treatment period. (C) Syngeneic tumor volume measured every 3

days; data are presented as tumor growth curves over 27 days. (D) Representative photographs of syngeneic tumors at the study endpoint (upper); endpoint tumor weight for all groups (lower). (E–F) Quantification of O-GlcNAcylation and PGC-1α expression levels was performed using ImageJ software on IHC images. (G) Levels of cell migration-related proteins E-cadherin and Vimentin in tumor lysates were detected by WB. Data are mean ± SD, n = 5. *, $P < 0.05$ versus control; #, $P < 0.05$ versus the indicated group.

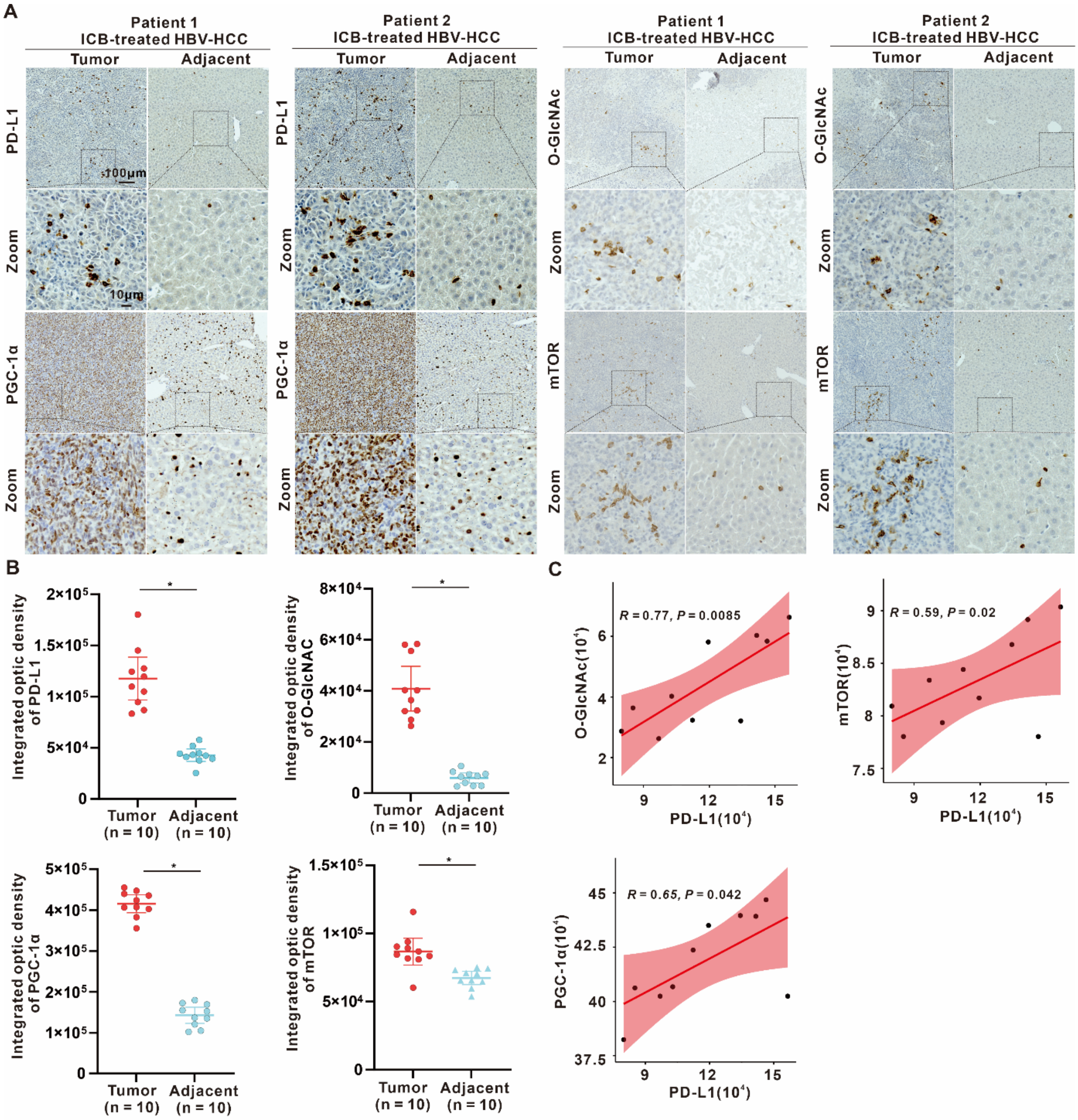

Fig. S10. PD-L1 is upregulated in ICB-treated HBV-related HCC and positively correlates with global O-GlcNAcylation, mTOR, and PGC-1α in paired clinical specimens. (A)

Representative IHC staining of PD-L1, global O-GlcNAcylation, PGC-1α, and mTOR in ICB-treated HBV-HCC tissues and paired adjacent tissues (n = 10 pairs). Scale bar: 100 μm (main) and 10 μm (Zoom). (B) Quantification of PD-L1, global O-GlcNAcylation, PGC-1α, and mTOR expression levels was performed using ImageJ software on IHC images. (C) Pearson correlation analysis of PD-L1 expression with O-GlcNAcylation, PGC-1α, and mTOR protein expression in tumor tissues. Correlation coefficients (*R*) and *P* values are indicated. Data are mean ± SD, n = 10 pairs. *, $P < 0.05$ versus adjacent tissue.

Supplementary tables

Table S1. Information on GEO datasets used in the present study

Dataset IDs	Omics types	Total samples
GSE147888	Microarray	24
GSE251942	RNA-Seq	25
GSE282701	RNA-seq	12
GSE269528	RNA-Seq	6
GSE94660	Microarray	42
GSE65485	RNA-seq	55
GSE36376	Microarray	433
GSE87410	RNA-seq	100
GSE74656	Microarray	15
GSE84598	Microarray	66
GSE63898	Microarray	138
GSE14811	Microarray	112
GSE29721	Microarray	20
GSE105130	RNA-seq	52
GSE169289	RNA-seq	34
GSE135631	RNA-seq	30
GSE76297	Microarray	304
GSE59259	Microarray	16
GSE15654	Microarray	216
GSE54236	Microarray	161
GSE25097	Microarray	401
GSE84402	Microarray	28
GSE57555	Microarray	64

Table S2. Details of primary antibodies used for immunohistochemistry in the present study

Antibodies	Resources	Dilution
Anti-PD-L1	ABclonal	1:200
Anti-PGC-1α	Affinity	1:200
Anti-TFAM	Affinity	1:200
Anti-NRF1	Affinity	1:200
Anti-PCNA	Santa Cruz	1:200
Anti-Ki67	ABclonal	1:200
Anti-O-GlcNAc (CTD110.6)	Santa Cruz	1:200

Table S3. Details of primary and secondary antibodies used for immunofluorescence in the present study

Antibodies	Resources	Dilution
Anti-GOLPH3	ABclonal	1:200
Anti-PD-L1	ABclonal	1:200
Anti-TOM20	ABclonal	1:200
Anti-GZMB	Abmart	1:200
Anti-CD8α	Santa Cruz	1:200
Anti-Pan-CK	ABclonal	1:200
Alexa Fluor 488-labeled anti-rabbit/mouse	Beyotime	1:400
Alexa Fluor 647-labeled anti-rabbit/mouse	Beyotime	1:400

Table S4. Primer sequences used in the present study

Gene symbol	Primer sequences (5'→3')
ACTB (Human)	FP: 5'-CATGTACGTTGCTATCCAGGC-3'<br>RP: 5'-CTCCTTAATGTCACGCACGAT-3'
CD274 (Human)	FP: 5'-TGGCATTGCTGAACGCATTT-3'<br>RP: 5'-TGCAGCCAGGTCTAATTGTTTT-3'
CYTB (Human)	FP: 5'-AACTTCGGCTCACTCCTTGG-3'<br>RP: 5'-GGAGGTGATTCCTAGGGGGT-3'
ATP6 (Human)	FP: 5'-CCTAGGACCGCAGGTTTTAT-3'<br>RP: 5'-GCGGTTGCTAGGAAGAGTTG-3'
ATP8 (Human)	FP: 5'-CCTTCCTCACTACTCACTCCTC-3'<br>RP: 5'-TGAGGAAGTAGGAGAGGATGAG-3'
ND5 (Human)	FP: 5'-CAGGCCCATTAAGAGCGTAA-3'<br>RP: 5'-TGGAGGGTTGGTTGGTCTTA-3'
PPARGC1A (Human)	FP: 5'-TCTGAGTCTGTATGGAGTGACAT-3'<br>RP: 5'-CCAAGTCGTTCACATCTAGTTCA-3'
TFAM (Human)	FP: 5'-ATGGCGTTTCTCCGAAGCAT-3'<br>RP: 5'-TCCGCCCTATAAGCATCTTGA-3'
NFE2L1 (Human)	FP: 5'-AGTGGAGGAGGACAGAGGAAG-3'<br>RP: 5'-TTCTTCCTGGGTTGAGGTTG-3'
sg*CD274*#1 (Human)	FP: 5'-CACCGCTGAACGCATTTACTGTCA-3'<br>RP: 5'-AAACTGACAGTAAATGCGTTCAGC-3'
sg*CD274*#2 (Human)	FP: 5'-CACCGCATAGTAGCTACAGACAGA-3'<br>RP: 5'-AAACTCTGTCTGTAGCTACTATGC-3'
sg*CD274*#3 (Human)	FP: 5'-CACCGACAGCTGAATTGGTCATCCC-3'<br>RP: 5'-AAACGGGATGACCAATTCAGCTGTC-3'

Table S5. Details of primary and secondary antibodies for western blotting in the present study

Antibodies	Resources	Dilution
Anti-β-actin	ABclonal	1:5000
Anti-HBx	Santa Cruz	1:500
Anti-COX IV	Santa Cruz	1:500
Anti-Na^+/K^+-ATPase	ABclonal	1:1000
Anti-GOLPH3	ABclonal	1:1000
Anti-Drp1	ABclonal	1:1000
Anti-PD-L1	ABclonal	1:1000
Anti-TOM20	ABclonal	1:1000
Anti-mTOR	ABclonal	1:1000
Anti-p-$mTOR^{Ser2448}$	ABclonal	1:1000
Anti-PGC-1α	Affinity	1:1000
Anti-E-cadherin	ABclonal	1:1000
Anti-Vimentin	ABclonal	1:1000
Anti-TFAM	Affinity	1:1000
Anti-NRF1	Affinity	1:1000
Anti-OGT	ABclonal	1:1000
Anti-O-GlcNAc (CTD110.6)	Santa Cruz	1:500
Anti-Cleaved-Caspase 3	Affinity	1:1000
Anti-BAX	Affinity	1:1000
Anti-Bcl-2	Santa Cruz	1:500
HRP-conjugated rabbit anti-human IgG1 (Fc) mAb	ABclonal	1:2000
HRP-conjugated mouse anti-human IgG1 (Fc) mAb	ABclonal	1:2000
